# Light and Primary Production Shape Bacterial Activity and Community Composition of Aerobic Anoxygenic Phototrophic Bacteria in a Microcosm Experiment

**DOI:** 10.1128/mSphere.00354-20

**Published:** 2020-07-01

**Authors:** Kasia Piwosz, Ana Vrdoljak, Thijs Frenken, Juan Manuel González-Olalla, Danijela Šantić, R. Michael McKay, Kristian Spilling, Lior Guttman, Petr Znachor, Izabela Mujakić, Lívia Kolesár Fecskeová, Luca Zoccarato, Martina Hanusová, Andrea Pessina, Tom Reich, Hans-Peter Grossart, Michal Koblížek

**Affiliations:** a Center Algatech, Institute of Microbiology, Czech Academy of Sciences, Třeboň, Czechia; b Institute of Oceanography and Fisheries, Split, Croatia; c Department of Aquatic Ecology, Netherlands Institute of Ecology (NIOO-KNAW), Wageningen, The Netherlands; d Great Lakes Institute for Environmental Research, University of Windsor, Windsor, Ontario, Canada; e University Institute of Water Research, University of Granada, Granada, Spain; f Marine Research Centre, Finnish Environment Institute, Helsinki, Finland; g Department of Natural Sciences, University of Agder, Kristiansand, Norway; h Israel Oceanographic and Limnological Research, National Center for Mariculture, Eilat, Israel; i Institute of Hydrobiology, Biology Centre, Czech Academy of Sciences, České Budějovice, Czechia; j Department Experimental Limnology, Leibniz Institute of Freshwater Ecology and Inland Fisheries (IGB), Stechlin, Germany; k Department of Life and Environmental Sciences (DiSVA), Università Politecnica delle Marche, Ancona, Italy; l Haifa University, Haifa, Israel; m Institute of Biochemistry and Biology, Potsdam University, Potsdam, Germany; University of Wisconsin—Madison

**Keywords:** phytoplankton-bacteria coupling, aerobic anoxygenic phototrophic bacteria, bacterial community composition, AAP community composition

## Abstract

Phytoplankton is a key component of aquatic microbial communities, and metabolic coupling between phytoplankton and bacteria determines the fate of dissolved organic carbon (DOC). Yet, the impact of primary production on bacterial activity and community composition remains largely unknown, as, for example, in the case of aerobic anoxygenic phototrophic (AAP) bacteria that utilize both phytoplankton-derived DOC and light as energy sources. Here, we studied how reduction of primary production in a natural freshwater community affects the bacterial community composition and its activity, focusing primarily on AAP bacteria. The bacterial respiration rate was the lowest when photosynthesis was reduced by direct inhibition of photosystem II and the highest in ambient light condition with no photosynthesis inhibition, suggesting that it was limited by carbon availability. However, bacterial assimilation rates of leucine and glucose were unaffected, indicating that increased bacterial growth efficiency (e.g., due to photoheterotrophy) can help to maintain overall bacterial production when low primary production limits DOC availability. Bacterial community composition was tightly linked to light intensity, mainly due to the increased relative abundance of light-dependent AAP bacteria. This notion shows that changes in bacterial community composition are not necessarily reflected by changes in bacterial production or growth and vice versa. Moreover, we demonstrated for the first time that light can directly affect bacterial community composition, a topic which has been neglected in studies of phytoplankton-bacteria interactions.

**IMPORTANCE** Metabolic coupling between phytoplankton and bacteria determines the fate of dissolved organic carbon in aquatic environments, and yet how changes in the rate of primary production affect the bacterial activity and community composition remains understudied. Here, we experimentally limited the rate of primary production either by lowering light intensity or by adding a photosynthesis inhibitor. The induced decrease had a greater influence on bacterial respiration than on bacterial production and growth rate, especially at an optimal light intensity. This suggests that changes in primary production drive bacterial activity, but the effect on carbon flow may be mitigated by increased bacterial growth efficiencies, especially of light-dependent AAP bacteria. Bacterial activities were independent of changes in bacterial community composition, which were driven by light availability and AAP bacteria. This direct effect of light on composition of bacterial communities has not been documented previously.

## Abstract (precis)

Metabolic coupling between phytoplankton and bacteria determines the fate of dissolved organic carbon in aquatic environments, and yet how changes in the rate of primary production affect the bacterial activity and community composition remains understudied. Here, we experimentally limited the rate of primary production either by lowering light intensity or by adding a photosynthesis inhibitor. The induced decrease had a greater influence on bacterial respiration than on bacterial production and growth rate, especially at an optimal light intensity. This suggests that changes in primary production drive bacterial activity, but the effect on carbon flow may be mitigated by increased bacterial growth efficiencies, especially of light-dependent AAP bacteria. Bacterial activities were independent of changes in bacterial community composition, which were driven by light availability and AAP bacteria. This direct effect of light on composition of bacterial communities has not been documented previously.

## INTRODUCTION

The strength and nature of the coupling between phytoplankton and heterotrophic bacteria largely determines the fate of dissolved organic carbon (DOC) in aquatic environments. At times when primary production is insufficient to meet bacterial carbon demand, bacterial communities shift to utilize allochthonous carbon sources derived from the terrestrial surrounding (1, 2). The composition of aquatic DOC is complex and as yet poorly described (3). Moreover, most of the DOC in lakes is refractory, and semilabile and labile fractions contribute solely one-fourth to the total DOC pool (4). Phytoplankton primary production represents the main source of labile and semilabile carbon in the majority of large lakes (5, 6), where bacteria can utilize up to 40% of phytoplankton-derived DOC (7–10). Phytoplankton-derived DOC consists mainly of monomeric and combined carbohydrates, carboxylic acids, and amino acids, with minor contributions of other organic compounds like ketones, aldehydes, and even high-molecular-weight polymers (11).

Primary production and phytoplankton community composition also strongly influence bacterial community composition and affects their seasonal patterns in beta diversity (12, 13). Specific bacterial phylotypes, e.g., *Fluviicola* and *Limnohabitans*, are known to preferentially consume phytoplankton-derived DOC and thus to tightly follow phytoplankton dynamics throughout the season (14–16). On the other hand, correlation analysis has indicated that the direct effect of environmental factors may be more important than interactions with phytoplankton in shaping bacterial communities (17). To better understand the DOC dynamics in aquatic ecosystems, it is thus crucial to know the nature and strength of phytoplankton-bacteria interactions in regard to changing environmental conditions.

Various environmental factors, such as temperature, turbulence, nutrient concentrations and light, shape the fate of phytoplankton-bacteria interactions. Shifts from carbon commensalism to competition for inorganic nutrients are often observed when nutrients availability becomes limited (18–21). The effect of light is, on the other hand, usually considered to be indirect via phytoplankton-bacterial coupling (22), and studies on the direct effect typically focus on UV radiation (23). Nevertheless, direct effects of light on bacterial communities have also been suggested to exist (21, 24) and may be mediated by physiological responses of photoheterotrophic bacteria, such as rhodopsin-containing bacteria or aerobic anoxygenic phototrophic (AAP) bacteria. These organisms utilize energy from light for ATP synthesis but require organic carbon for growth (25). AAP bacteria produce ATP on bacteriochlorophyll-containing reaction centers in the process of cyclic photophosphorylation (25), and this additional energy allows them to reduce the requirement for oxidative phosphorylation (respiration), thus increasing their growth efficiency (26, 27). AAP bacteria represent the metabolically more active part of aquatic bacterial communities, presumably consuming a large fraction of phytoplankton-derived DOC (28, 29). For example, the *Limnohabitans* and *Polynucleobacter* lineages contain numerous AAP bacterial species (30, 31). In addition, seasonal maximum abundance of AAP bacteria follows that of phytoplankton blooms in freshwater lakes (32, 33). These observations suggest that AAP bacteria can represent a key functional bacterial group whose activity is strongly linked to primary production. The coupling between bacteria and phytoplankton, and hence the carbon flow through the microbial loop, is predicted to increase with climate warming and eutrophication (34). Therefore, it is important to elucidate how changes in primary production affect bacterial activities and community composition and thus the flow of nutrients and organic carbon through the ecosystem.

To test this, we reduced primary production rate within a native freshwater microbial community (Římov Reservoir, Czechia), either directly by inhibiting photosynthesis via a chemical inhibitor Diuron or indirectly by limiting the intensity of photosynthetically active radiation (PAR) available to phytoplankton. We hypothesized that a sudden decrease in primary production will lower bacterial activity and will shift the bacterial community toward groups with a lower dependence on phytoplankton-derived DOC. We also expected the dynamics of AAP bacteria to depend on light intensity. Bacterial activity changed in accordance with our prediction, but these changes did not seem to have resulted from differences in the bacterial community composition. Interestingly, bacterial community composition appeared to be directly affected by light intensity, and its changes were driven by an increase in the relative abundances of AAP bacteria.

## RESULTS

### Primary production and phytoplankton.

**FIG 1:**
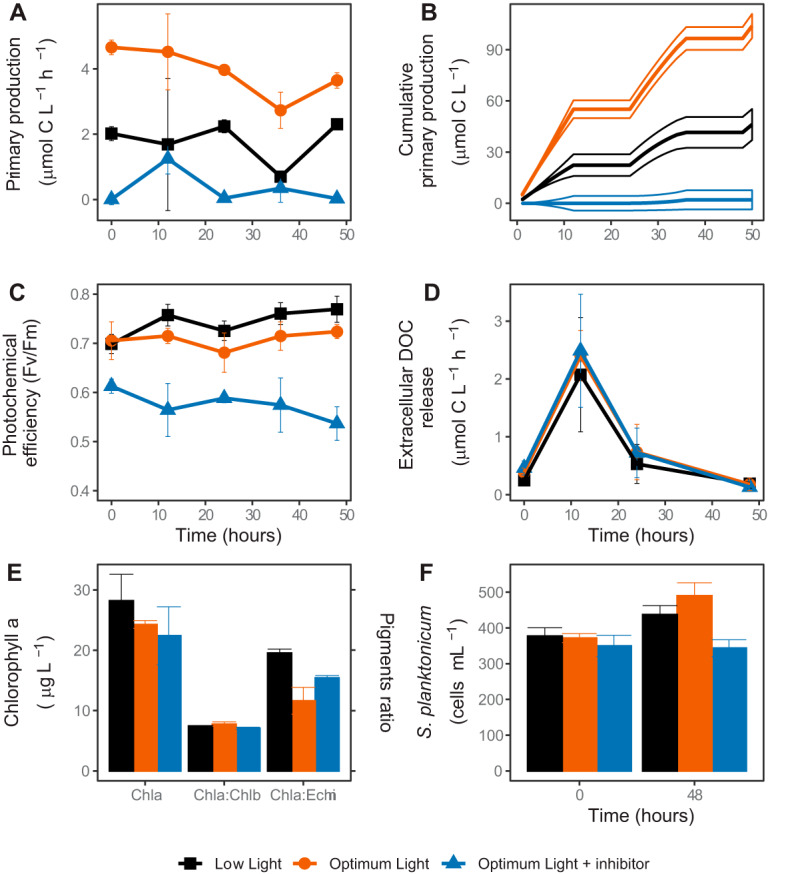
Phytoplankton activity and dynamics in the experimental treatments. (A) Rate of CO_2_ fixation (primary production); (B) cumulative CO_2_ fixation; (C) phytoplankton community photosynthetic yield; (D) extracellular release of the fixed CO_2_; (E) concentrations of chlorophyll *a* (Chl*a*), ratios of Chl*a* to chlorophyll *b* (Chl*a*:Chl*b*, indicative of chlorophyte algae), and ratios of Chl*a* to echinenone (Chl*a*:Echin, indicative of cyanobacteria) at the end of the experiment, (F) abundance of *Staurastum planktonicum* at the beginning and the end of the experiment.

The measurements of carbon fixation rates documented that the experimental manipulation of primary production was successful. Primary production differed significantly between the treatments (*P* < 0.001) and did not change in any of the treatments throughout the experiment (*P* > 0.060, Fig. 1A). Time-integrated primary production at the end of the experiment was over two times higher in the control treatment at optimum light (OL; PAR, ∼200 μmol photons m^−2^ s^−1^; average time-integrated primary production, 96.6 μmol C liter^−1^; 95% confidence interval, 89.9 to 103.3) than in the low-light treatment (LL; PAR, ∼35 μmol photons m^−2^ s^−1^; 41.5 μmol C liter^−1^; 32.5 to 50.6), and it was very low in the treatment at the optimum light intensity with photosynthesis inhibited chemically (OL-Inh; 2.0 μmol C liter^−1^; 3.5 to 7.6; Fig. 1B). The relative photochemical yield was similar in both OL and LL treatments (*P* = 0.139) but was significantly lower in the OL-Inh treatment (*P* < 0.001, Fig. 1C). Differences in extracellular DOC release between the treatments were not significant (*P* = 0.780, Fig. 1D).

Furthermore, we did not observe any significant differences in the chlorophyll *a* (Chl-*a*) concentration (*P* = 0.330) or Chl-*a*/Chl-*b* ratios (*P* = 0.051) between the treatments at the end of the experiment (Fig. 1E). However, the abundance of the dominant phytoplankton species, *Staurastrum planktonicum*, was significantly lower in the OL-Inh treatment than in the OL (*P* < 0.001) and LL (*P* = 0.032) treatments (Fig. 1F). Moreover, the Chl-*a*/echinenone (carotenoid specific to cyanobacteria) ratio was significantly higher in the LL treatment than in the OL treatment (*P* = 0.011, Fig. 1E), indicating a shift in the phytoplankton community composition.

### Total bacterial and AAP bacterial abundance and activity.

**FIG 2:**
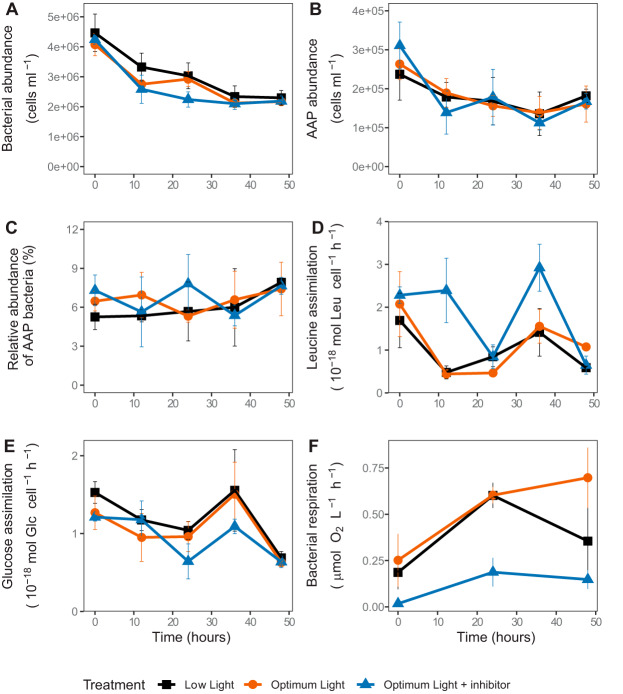
Bacterial activity and dynamics in the experimental treatments. (A) Abundance of all bacteria; (B) abundance of AAP bacteria; (C) relative abundance of AAP; (D) specific assimilation rate of leucine; (E) specific assimilation rate of glucose; (F) bacterial community respiration.

Total bacterial abundance declined during the experiment but did not differ between the treatments (*P* = 0.391, Fig. 2A). Similar trends were observed for AAP bacterial abundance (Fig. 2B), whose contribution to the total bacterial abundance did not change throughout the experiment (*P* > 0.371, Fig. 2C).

The initial specific assimilation rate of leucine, an indicator of bacterial protein and biomass production, was reduced by half within the first 12 h in the LL and OL treatments (Fig. 2D). In contrast, in the OL-Inh treatment this rate decreased only after 24 h and increased again to 2.98 ± 0.5 × 10^−18^ mol Leu cell^−1^ h^−1^ at 36 h. Nevertheless, specific assimilation rates of leucine were similar in all treatments at the end of the experiment (Fig. 2D). Bacterial growth rates, calculated based on leucine assimilation, varied from 0.17 ± 0.05 day^−1^ to 0.61 ± 0.22 day^−1^ (average ± the standard deviations [SD]) in the LL treatment, from 0.17 ± 0.01 day^−1^ to 0.75 ± 0.27 day^−1^ in the OL treatment and from 0.23 ± 0.07 day^−1^ to 0.96 ± 0.35 day^−1^ in the OL-Inh treatment. The enzymatic activity of leucine aminopeptidase (LAPase) was similar in all treatments throughout the experiment indicating indifferent requirements for this amino acid (see Fig. S1A in the supplemental material).

10.1128/mSphere.00354-20.3 FIG S1 (A) Enzymatic activity of leucine aminopeptidase (normalized to abundance of high nucleic acid content [HNA bacteria]); (B) enzymatic activity of beta-glucosidase (normalized to abundance of HNA bacteria); (C) C:N molar ratios in particulate organic matter (POM); (D) C:P molar ratios in POM; (E) N:P molar ratios in POM; (F) enzymatic activity of alkaline phosphatase (normalized to the concentration of Chl*a*). Download FIG S1, PDF file, 0.01 MB. Copyright © 2020 Piwosz et al. 2020 Piwosz et al. This content is distributed under the terms of the Creative Commons Attribution 4.0 International license.

The specific assimilation rate of glucose, a molecule mainly used for energy metabolisms and ATP production, was similar in all treatments, and did not vary substantially throughout the experiment (Fig. 2E). Enzymatic activity of β-1,4-glucosidase (βGase) accelerated in the OL and LL treatments and differed significantly between the OL and OL-Inh treatments (*P* = 0.011, Fig. S1B), indicating a lower uptake of glucose in the OL-Inh treatment.

Bacterial respiration increased within the first 24 h in both OL and LL treatments but continued to increase during the subsequent 24 h only in the OL treatment (Fig. 2F). Bacterial respiration was significantly lower in the OL-Inh treatment (*P* = 0.012). The total primary production satisfied about 38% ± 13% of the total bacterial carbon demand at the beginning of the experiment, and this proportion did not differ between the treatments (*P* = 0.134). It decreased throughout the experiment to 22% ± 8% in the LL treatment, 14% ± 6% in the OL treatment, and 0.5% ± 0.4% the OL-Inh treatment. The difference between the LL and OL-Inh treatments was significant (*P* < 0.017), indicating lower carbon availability for bacteria due to photosynthetic inhibition.

**FIG 3:**
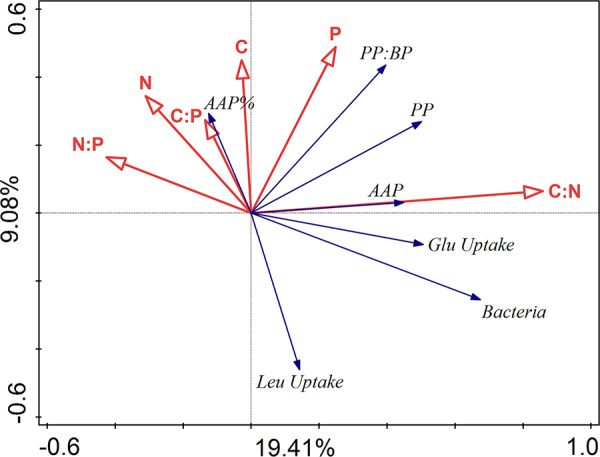
Redundancy analysis (RDA) correlation biplot using the biotic factors as response variables (blue arrows) and environmental (chemical) factors as explanatory variables (red arrows). Correlations between variables are indicated by the angle between arrows (an angle <90° between two arrows of interest implies positive correlation, a value equal to 90° implies lack of any correlation, and >90° implies negative correlations), whereas the length of an arrow depicts the strength of association between a variable and the ordination axes shown in the biplot. The proportions of the total variability explained by the first two axes are given. The total variance explained by the explanatory variables was 34%. Labeling: Bacteria, cell counts (per ml) of heterotrophic bacteria; AAP, cell counts (per ml) of aerobic anoxygenic phototrophic bacteria; PP, primary production; BP, bacterial production; Leu Uptake, specific assimilation rate of leucine; Glu Uptake, specific assimilation rate of glucose uptake; C, particulate organic carbon; N, particulate organic nitrogen; P, particulate organic phosphorus.

To elucidate the relationship between bacterial numbers and activity, as well as pools of particulate organic carbon (POC), particulate organic nitrogen (PON), and particulate organic phosphorous (POP), we performed a redundancy analysis (RDA) (Fig. 3). The explanatory variables accounted for 34% of the total variance in the picoplankton, with the first two RDA axes explaining 28.50% of the variance. The key explanatory variables were POC:PON ratio (explaining 14.6%, pseudo-F = 6.8, *P* = 0.002) and PON:POP ratio (explaining 5.2%, pseudo-F = 2.2, *P* = 0.046). Total bacterial and AAP bacterial abundance and specific assimilation rates of leucine and glucose correlated strongly with POC:PON ratio, and negatively with PON:POP ratio and concentration of PON, while the percent contribution of AAP bacteria to total bacterial abundance was strongly correlated with POC:POP ratio (Fig. 3).

### Bacterial community composition.

Rarefaction analysis indicated that a sequencing depth of 25,000 reads was sufficient to cover for most of the bacterial diversity (Fig. S2A-C). *Actinobacteriota*, *Bacteroidota*, *Proteobacteria*, and *Verrucomicrobiota* dominated in all treatments, while the relative abundance of both *Proteobacteria* and *Verrucomicrobiota* increased most throughout the experiment (Fig. S3A). *Proteobacteria* were initially dominated by the orders *Acetobacterales* and *Burkholderiales* (Fig. S3B). The relative abundance of *Burkholderiales* showed a subsequent decrease, which was accompanied by an increase in the relative abundance of *Caulobacterales* and *Rhizobiales*. *Verrucomicrobiota* were dominated by the order *Chthoniobacterales*, whose relative abundance decreased after 48 h in OL and OL-Inh treatments, while *Pedosphaerales* and *Verrucomicrobiales* increased (Fig. S3C). Other phyla showed little changes throughout the experiment (Fig. 3D to F).

10.1128/mSphere.00354-20.4 FIG S2 Rarefaction curves for each treatment for 16S rRNA (left panel) and *pufM* (right panel) gene amplicons. (A and D) LL treatment; (B and E) OL treatment; (C and F) OL-Inh treatment. T with a number denotes a time point, and the letters A to C indicate replicates. Download FIG S2, PDF file, 0.3 MB. Copyright © 2020 Piwosz et al. 2020 Piwosz et al. This content is distributed under the terms of the Creative Commons Attribution 4.0 International license.

10.1128/mSphere.00354-20.5 FIG S3 Composition of bacterial communities in the experimental treatments (based on the 16S rRNA gene amplicons). (A) Percent contribution of phyla to the total number of reads in the sequencing libraries; (B) percent contribution of orders to the number of reads coming from *Proteobacteria*; (C) percent contribution of orders to the number of reads coming from *Verrucomicrobiota*; (D) percent contribution of orders to the number of reads coming from *Planctomycetota*; (E) percent contribution of orders to the number of reads coming from *Actinobacteriota*; (F) percent contribution of orders to the number of reads coming from *Bacteroidota*. NA, unclassified. Download FIG S3, PDF file, 0.4 MB. Copyright © 2020 Piwosz et al. 2020 Piwosz et al. This content is distributed under the terms of the Creative Commons Attribution 4.0 International license.

**FIG 4:**
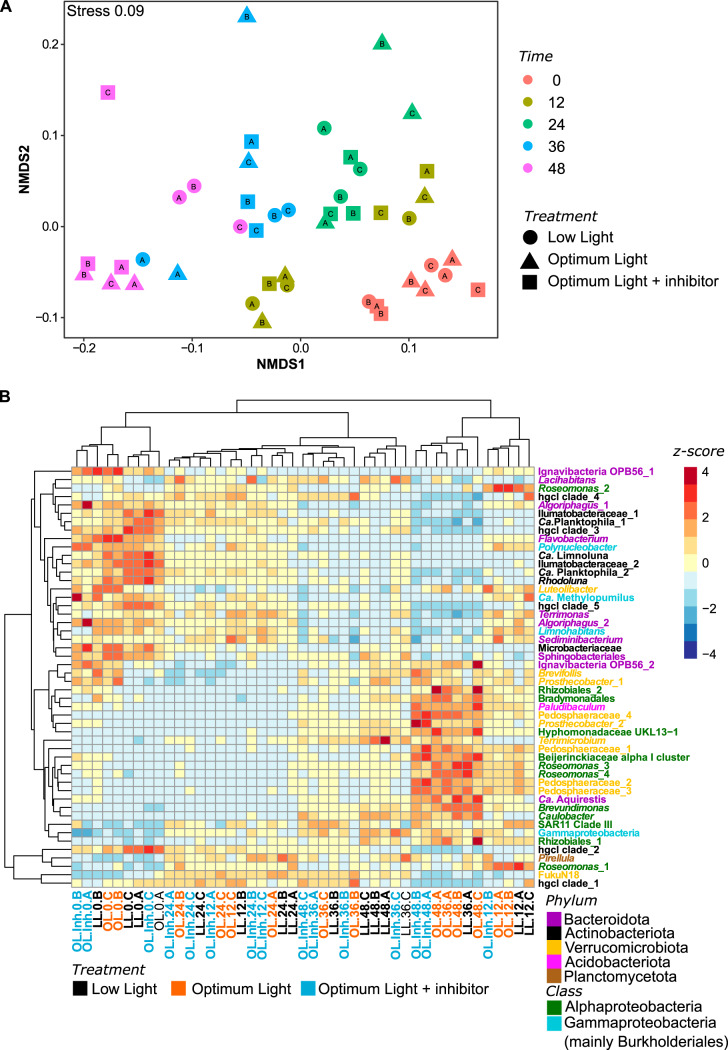
Changes in total bacterial communities during the experiment based on the 16S rRNA amplicons. (A) Nonmetric multidimensional scaling plot showing changes in beta diversity based on Bray-Curtis distances; (B) heatmap showing changes in the relative abundance of reads of 50 most abundant ASVs. Blue represents a low and red represents a high contribution of an ASV. Clustering was done using the unweighted pair group method with arithmetic mean (UPGMA) method on Bray-Curtis distances calculated from the percent data. The values were centered and scaled by removing the mean and then dividing by the standard deviations to facilitate visualization of both abundant and rare ASVs. Bacterial phyla are color-coded as indicated in the legend. LL, low light treatment; OL, optimum light treatment; OL-Inh, optimum light with inhibitor treatment. Numbers after the treatment abbreviations indicate time points; the A to C suffixes indicate replicates.

The bacterial communities at the end of the experiment were very similar in the OL and OL-Inh treatments (Fig. 4A), and they differed significantly from that in the LL treatment (*P* = 0.029). The difference could be attributed to a decrease in the relative abundances of amplicon sequence variants (ASVs) affiliated with *Actinobacteriota* (such as “*Candidatus* Planktophila,” *Ca*. Rhodoluna,” and “*Ca*. Limnoluna”), *Bacteroidota* (such as *Lacihabitans*, *Terrimonas*, *Algoriphagus*, and *Sediminibacterium*), and *Gammaprotebacteria* (such as *Limnohabitans* and *Polynucleobacter*) and a concomitant increase of the ASVs affiliated with *Alphaproteobacteria* (such as *Roseomonas*, *Rhizobiales*, *Brevundimonas*, *Caulobacter*, and *Hyphomonadaceae*), *Verrucomicrobiota* (*Brevifollis*, *Terrimicrobium*, *Prosthecobacter*, and *Pedosphaeraceae*), and *Planctomycetota* in OL and OL-Inh treatments (Fig. 4B).

### AAP bacterial community composition.

The rarefaction analysis indicated that the sequencing depth of 50,000 reads was sufficient to cover for most of the diversity of AAP bacterial communities in the majority of samples (Fig. S2D to F).

AAP bacteria were represented by three classes: *Alphaproteobacteria*, *Gammaproteobacteria*, and *Gemmatimonadetes* (phylum *Gemmatimonadota*). *Gemmatimonadetes* remained relatively stable during the experiment representing on average 4.2% ± 0.9% of reads in all libraries. The main change in all treatments was the increase in the relative abundance of *Alphaproteobacteria* (from 26.1% ± 5.1% to 54.5% ± 5.6%) and the concomitant decrease in *Gammaproteobacteria* (from 70.8% ± 5.9% to 40.7% ± 5.6%; Fig. S4A). The main orders were *Rhodobacterales* (*Alphaproteobacteria*) and *Burkholderiales* (*Gammaproteobacteria*). The relative abundance of *Rhodobacterales* increased ∼3-fold throughout the experiment in all treatments (Fig. S4B), mainly due to the increase of the *Hyphomonadaceae* family (Fig. S4C). In contrast, *Burkholderiales* initially dominated the AAP bacterial community in all treatments (about 70% of reads), but their relative abundance decreased to 43% ± 0.9% in the LL treatment, 31% ± 0.4% in the OL treatment, and 36% ± 6.1% in the OL-Inh treatment (Fig. S4B). The relative abundance of *Burkholderiales*’ dominant genera, *Limnohabitans* and *Polynucleobacter*, decreased throughout the experiment in all treatments, while the abundance of *Rhizobacter* and *Methylibium* increased (Fig. S4D).

10.1128/mSphere.00354-20.6 FIG S4 Composition of AAP bacteria communities in the experimental treatments (based on the *pufM* amplicons). (A) Percent contribution of classes to the total number of reads in the sequencing libraries; (B) percent contribution of proteobacterial orders to the proteobacterial number of reads; (C) percent contribution of families within *Rhodobacteriales* order; (D) percent contribution of classified genera within *Burkholderiales* order. NA, unclassified. Download FIG S4, PDF file, 0.2 MB. Copyright © 2020 Piwosz et al. 2020 Piwosz et al. This content is distributed under the terms of the Creative Commons Attribution 4.0 International license.

**FIG 5:**
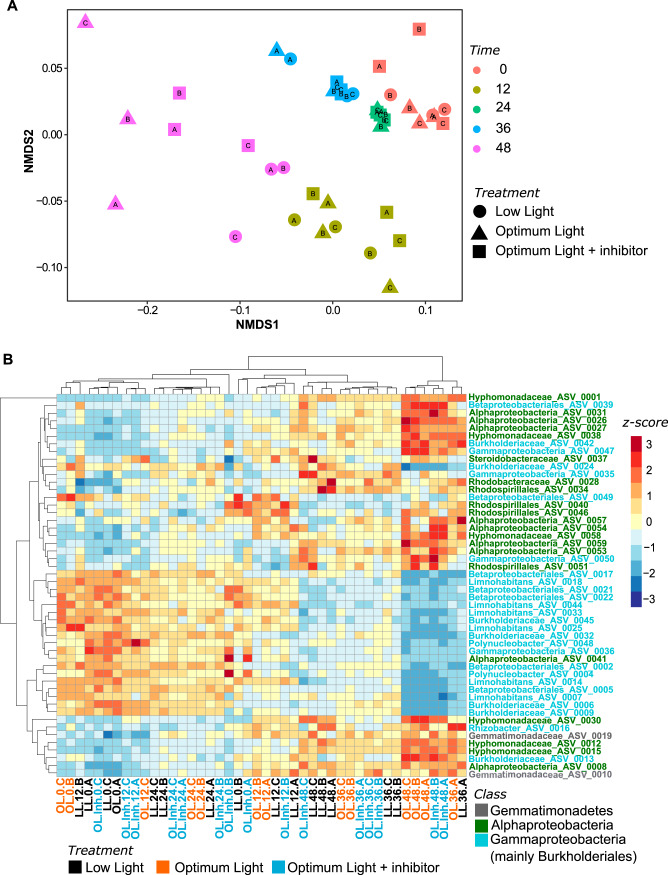
Changes in AAP bacterial communities during the experiment based on the *pufM* amplicons. (A) Nonmetric multidimensional scaling plot showing changes in beta-diversity based on Bray-Curtis distances; (B) heatmap showing changes in the relative abundance of reads of 50 most abundant ASVs. Blue represents a low and red represents a high contribution of an ASV. Clustering was done using the unweighted pair group method with arithmetic mean (UPGMA) method on Bray-Curtis distances calculated from the percent data. The values were centered and scaled by removing the mean and then dividing by the standard deviations to facilitate visualization of both abundant and rare ASVs. Bacterial phyla are color-coded as indicated in the legend. LL, low light treatment; OL, optimum light treatment; OL-Inh, optimum light with inhibitor treatment. Numbers after the treatment abbreviations indicate time points; the A to C suffixes indicate replicates.

At the end of the experiment, the community of AAP bacteria in the LL treatment differed significantly (*P* = 0.021) from that in the OL treatment (Fig. 5A). This could be attributed to a greater increase in the relative abundance of ASV affiliated with *Alphaproteobacteria* in the OL treatment compared to the LL treatment (Fig. 5B).

## DISCUSSION

The key challenge of our study was to modify the rate of primary production without adding inorganic nutrients, which could also alter the nature of phytoplankton-bacteria interactions, e.g., from carbon commensalism to competition for inorganic nutrients (18–20, 35, 36). Therefore, we manipulated primary production either by decreasing irradiance or by adding the specific photosystem II inhibitor Diuron (37). The concentration of soluble reactive phosphorus at the start of the study (Table S1) was in a range that limits phytoplankton growth in the Římov Reservoir, from which the microbial community was sampled (38, 39). To avoid severe nutrient limitation, the incubations were restricted to 48 h. The increased molar ratios of POC:POP (>90) and PON:POP (>13), an elevated level of alkaline phosphatase (APase) activity (Fig. S1C to F), and an association of primary production with POP (Fig. 3) suggest that the phytoplankton was P limited. Nevertheless, primary production and relative photochemical yield remained unaffected throughout the incubation time in each treatment (Fig. 1A and C). Consequently, our experiment was characterized by well-defined environmental settings with clearly modified primary production rates between the different treatments, whereas all other environmental conditions remained relatively unaffected. This allowed direct investigation in a “quasinatural setting” of the effect of primary production versus light on bacterial activity and community composition.

10.1128/mSphere.00354-20.2 TABLE S1 Physical, chemical, and biological conditions in the Římov Reservoir on 21 August 2017. Download Table S1, PDF file, 0.4 MB. Copyright © 2020 Piwosz et al. 2020 Piwosz et al. This content is distributed under the terms of the Creative Commons Attribution 4.0 International license.

### Effect of changes in primary production on total bacterial activity and phytoplankton-bacteria coupling.

The induced changes in primary production affected bacterial respiration according to our hypothesis, with the highest oxygen consumption rates when primary production was highest (OL treatment), and the lowest when primary production was inhibited (OL-Inh treatment; Fig. 1A and 2F). This indicates a direct bacterial dependence on phytoplankton-derived carbon, providing a possible explanation for a strong correlation between primary and bacterial production observed in freshwater lakes (7). The very low respiration rates in the OL-Inh treatment indicate low carbon availability for bacteria, since labile DOC (e.g., glucose) derived from phytoplankton fuels mainly bacterial respiration (2, 10). Since there was no indication of increased phytoplankton death in our experiment (Fig. 1), active total primary production was the main source of carbon available for heterotrophic bacteria. Initially, it could satisfy 38% ± 13% of total bacterial carbon demand, which is within the range measured previously in the Římov Reservoir in summer (8). Throughout the experiment, however, this proportion decreased to almost null in the OL-Inh treatment, limiting the availability of photosynthetic carbon for bacteria. The inverse relation of the POC:POP ratio and the bacterial abundance and specific assimilation rates of leucine and glucose (Fig. 3) also indicate an overall low carbon availability (that is, the sum of DOC released extracellularly and fixed CO_2_) for bacteria in treatments with low primary production (40). Such conditions favor bacterial groups with higher growth efficiency, such as AAP bacteria (41). The competitive advantage of photoheterotrophic AAP bacteria over purely heterotrophic bacteria is enhanced with increasing irradiance, because AAP bacteria significantly reduce their respiration rates when producing ATP via energy absorbed on photosystems (26, 27). This may explain the higher bacterial growth rates in the OL-Inh treatment despite the lower carbon availability. Moreover, the stronger association between primary production and abundance of AAP bacteria compared to total bacterial abundance (Fig. 3) also suggests a tight relationship between AAP bacteria and phytoplankton. Taken together, these results show that a sudden decrease in primary production may trigger an almost immediate decline in bacterial activity, likely induced by carbon limitation to bacteria. However, this effect may be partially mitigated by the light-dependent ATP production by AAP bacteria, which therefore require less carbon in the light than purely heterotrophic bacteria (26, 27).

### Effect of change in primary production on bacterial communities.

Phytoplankton exudates are the main source of carbon for bacteria in the lacustrine parts of the Římov Reservoir (8). Changes in primary production and phytoplankton communities cause sudden growth of specific bacterial lineages, such as *Fluviiciola* sp. and *Limnohabitans* (14, 15), which are driven by compositional changes of phytoplankton derived DOC (16). Short-term blooms of these bacteria are subsequently terminated by strong protistan grazing (14) or viral infection (42). Protistan grazers and viruses were also present in our experiment, because applying size fractionation to exclude them would also remove primary producers or other species of our interest. Bacterial mortality was the highest during the first 12 h, but it decreased later on, and the bacterial abundance did not change within the last 12 h of the experiment; in the case of AAP bacteria it even increased (Fig. 2A and B). In contrast, changes in the total and AAP bacterial communities were significant only at the end of the experiment. Therefore, although the initial top-down pressure due to grazing or viral lysis on bacteria was high, this effect likely diminished throughout the experiment.

A general concern with experiments in enclosures is a “bottle effect,” which we intended to mitigate by an incubation time of <48 h (43). The high mortality rates (Fig. 2A) and the increase in the relative abundance of *Caulobacterales* (Fig. S4B) indicates that some “bottle effect” may have occurred. On the other hand, *Verrucomicrobiota* and *Alphaproteobacteria* (including the order *Caulobacterales*), which showed the highest increase in relative abundance throughout the experiment, were reported to be the most active groups based on 16S rRNA amplicon libraries constructed from the transcript in a mesocosm experiment conducted at the same time in the Římov Reservoir (44). Thus, although we were not able to completely exclude the “bottle effect” from our enclosures, we conclude that the observed changes in total bacterial and AAP communities resulted predominantly from their response to the manipulation of primary production and less from the “bottle effect” *per se*.

The bacterial communities changed most in the OL and OL-Inh treatments (Fig. 4A). The fact that they were similar in the treatments incubated at the same optimal light intensity, despite the differences in primary production rates, phytoplankton community composition, and phytoplankton-bacteria coupling (Fig. 1), suggests a direct effect of light on the bacterial community composition. In contrast, the changes in bacterial community composition were less conspicuous in the LL treatment (Fig. 4B), and it significantly differed from OL and OL-Inh treatments at the end of the experiment. The higher light intensity in OL and OL-Inh treatments compared to LL treatment could favor photoheterotrophic bacteria, such as AAP bacteria, whose growth efficiency has been documented to be enhanced by light both in the laboratory and in marine waters (26, 27, 41). In agreement with this assumption, proteobacterial ASVs in the total bacterial community affiliated with genera known to contain AAP bacterial species, such as *Limnohabitans*, *Polynucleobacter* (*Burkholderiales*), and *Roseomonas* (*Rhodospirillalles*) (31, 44), were also found to be relatively abundant members of the AAP community (Fig. 4B and 5B). Moreover, we observed a clear shift in the total and AAP bacteria community toward the dominance of *Alphaproteobacteria* in the well-illuminated (OL and OL-Inh) treatments compared to the LL treatment (Fig. 4B and 5B; Fig. S4A and 5A). This indicates that the growth of photoheterotrophic *Alphaproteobacteria* was more influenced by light than by changes in primary production *per se*. In contrast, responses of the photoheterotrophic *Burkholderiales* seem to be more affected by the direct interaction with phytoplankton (16, 45). Our study provides the first observation that irradiance can directly affect the composition of the AAP community even more than primary production, emphasizing the need for including measurements of photoheterotrophy in general bacterial measurements (46).

### Conclusions.

This study aimed to evaluate the direct effect of a sudden decrease in primary production on bacterial activity and community composition. Our main finding is that such change in primary production resulted in a greater reduction of bacterial respiration than of overall bacterial production and growth. This indicates that when primary production limits bacterial activity, the consequences on carbon flow to higher trophic levels can be mitigated by an increase in bacterial growth efficiency. In particular, AAP bacteria could exhibit a high growth efficiency at optimal light availability. These changes in bacterial activity seem to be independent of changes in composition of the overall bacterial community. Bacterial community composition appeared to be directly related to differences in light intensity, and the changes were largely driven by increase in relative abundance of AAP bacteria. The indirect effect of reduced primary production affected bacterial community composition only marginally. Thus, light must be regarded as an important variable that directly drives the microbial community composition independent of photosynthetically fixed carbon availability in a short time scale. This is the first study, to our knowledge, that can distinguish between effects of reduced light versus reduced primary production on bacterial activity and community composition.

## MATERIALS AND METHODS

### Sampling site and water sampling.

The Římov Reservoir is a dimictic, meso-eutrophic, canyon-shaped reservoir in the southern part of Czechia that was built in 1979 to store drinking water. The reservoir is 13.5 km long with a maximum depth of 43 m. The surface area is 2.06 km^2^, with a volume of 34.5 × 10^6^ m^3^, and the average retention time in the summer is 77 days (for details, see reference 47).

Water was collected from a 0.5-m depth at a regular sampling site near the dam (48.846°N 14.487°E) on 21 August 2017 in the afternoon using a Friedinger sampler. After 30 min of sampling, 200 liters of water prefiltered through a 200-μm mesh to remove zooplankton were carried in a 500-liter plastic barrel to the laboratory for further processing. The experiment was conducted during the 10th International Group for Aquatic Primary Productivity Meeting in Třeboň, Czechia (19 to 30 August 2017).

### Experimental design.

Eight-liter aliquots of the sampled water were distributed into nine clean polyethylene terephthalate bottles using Tygon tubing (Saint-Gobain, Courbevoie, France) and a peristaltic pump. We used a control treatment with optimum light intensity (here referred to as OL; PAR approximately 200 μmol photons m^−2^ s^−1^), based on photosynthesis-irradiance relationships measured on the natural phytoplankton community of the Římov Reservoir a week before the experiment (Fig. S5). We then added two treatments, in which primary production was inhibited either directly by inhibiting photosynthesis via the addition of a chemical substance, or indirectly by lowering light availability. To inhibit photosynthesis at the optimum light intensity (OL-Inh), Diuron [3-(3,4-dichlorophenyl)-1,1-dimethylurea] was added at final concentration of 10 μmol liter^−1^ (37). Diuron inhibits photosystem II in oxygenic phototrophs but does not inhibit AAP bacteria (48). The low light treatment (LL) was set at a PAR intensity of 35 μmol photons m^−2^ s^−1^. All treatments were performed in triplicates and incubated at the ambient water temperature (22°C) over a 12:12-h dark/light period. White light was provided by banks of Osram Dulux L 55W/865 (Osram, Munich, Germany) luminescent tubes with a spectral temperature of 6,500 K. Sampling started before the onset of the first light period after 12 h of the dark incubation and was conducted at 12-h intervals (before the onset and at the end of the light periods) for 48 h. Such short incubation time was chosen because we were interested in the short-term responses of the bacterial community, and we wanted to avoid effect of nutrient limitation in the enclosures. Subsamples for all measurements were taken simultaneously unless otherwise indicated. A detailed description of the sampling, experimental setup, and methods is provided in Text S1 in the supplemental material.

10.1128/mSphere.00354-20.7 FIG S5 Relationship between light intensity and oxygen production of natural microbial communities from Římov Reservoir measured 1 week before the experiment. Cyan points with error bars indicate the average value and standard deviations of triplicate measurements; the dashed line indicates the spline curve using the cubic equation. Download FIG S5, TIF file, 0.8 MB. Copyright © 2020 Piwosz et al. 2020 Piwosz et al. This content is distributed under the terms of the Creative Commons Attribution 4.0 International license.

### Phytoplankton abundances.

Samples for phytoplankton enumeration were taken at the beginning and the end of the experiment. They were preserved with acid Lugol’s solution and stored in the dark at room temperature. Cells of the desmid *Staurastum planktonicum*, which accounted for approximately 60% of the phytoplankton biomass in the Římov Reservoir at the time of the experiment (44), were counted with the Utermöhl method (49) using the microscope Olympus IMT1 (Olympus, Tokyo, Japan).

### Primary production.

Primary production was measured twice daily, at the beginning and at the end of the light period, using a slight modification of the ^14^C radiolabel method (50). The total activity of 1.85 kBq H^14^CO_3_ was added to 1-ml samples. For each incubation bottle, three sets of incubations were prepared for measuring (i) total carbon fixation, (ii) the fraction of primary production released as DOC, and (iii) dark CO_2_ assimilation. The samples were incubated next to the experimental units for 2 h in technical duplicates. After the incubations, the samples for the fraction of primary production released as DOC were gently filtered through a 0.2-μm polycarbonate filter into clean scintillation vials, and 100 μl of 1 mol liter^−1^ HCl was added to all vials to volatilize nonincorporated H^14^CO_3_. The vials were left for 24 h in an exhaust hood before 4 ml of scintillation liquid (Perkin-Elmer, Waltham, MA) was added. The activity was determined in a scintillation counter (Perkin-Elmer). The total dissolved inorganic carbon concentration (DIC) was calculated based on temperature, pH (Inolab pH 720; WTW Xylem, Inc., Rye Brook, NY), and alkalinity measurements (Metrohm 877; Herisau, Switzerland), and the total carbon fixation rate was calculated knowing the radiolabeled C uptake and the fraction of H^14^CO_3_ added to the total DIC pool.

### Pigment concentrations.

Seston from 0.6 to 1 liter of water was filtered onto GF/F glass fiber filters (Whatman, Plc., Maidstone, UK) at the end of the experiment. The filters were dried of excess water by gently pressing in a paper towel, and pigments were immediately extracted in acetone-methanol mixture (7:2 [vol/vol], high-pressure liquid chromatography [HPLC] grade; Penta, Prague, Czechia). Clear extracts were analyzed by using a Prominence-*i* HPLC system (Shimadzu, Kyoto, Japan) as described previously (44).

### Extracellular enzymatic activity.

Extracellular enzyme activities corresponding to alkaline phosphatase (APase; EC 3.1.3.1), β-1,4-glucosidase (βGase; EC 3.2.1.21), and leucine aminopeptidase (LAPase; EC 3.4.11.1) were measured according to general protocols (51). From each experimental replicate, technical triplicates were measured. Enzyme activities were calculated using reference standards prepared in 4 mmol liter^−1^ sodium bicarbonate. APase was normalized to Chl-*a* biomass whereas βGase and LAPase were normalized to cell abundance of bacteria with a high content of nucleic acid (HNA bacteria; see below).

### Particulate carbon, nitrogen, and phosphorus.

Particulate organic carbon (POC), nitrogen (PON), and phosphorus (POP) were determined by collecting 60 ml of seston on a prewashed GF/F filter (Whatman). Blank filters were also included.

POC and PON were analyzed on a FLASH 2000 organic elemental analyzer (Brechbueler, Inc., Interscience B.V., Breda, The Netherlands). POP was analyzed as described by Eaton and Franson (52) and Armstrong et al. (53) on a QuAAtro39 AutoAnalyzer (SEAL Analytical, Ltd., Southampton, UK).

### Total and AAP bacterial abundance.

Samples of 10 ml were fixed with sterile-filtered formaldehyde (Penta) to a final concentration of 1%, and 0.5 ml was filtered onto white polycarbonate filters (pore size, 0.2 μm; Nuclepore; Whatman). Cells were stained with DAPI (4′,6′-diamidino-2-phenylindole) at a concentration of 1 mg liter^−1^ (54). Total and AAP bacterial abundances were determined using an epifluorescence Zeiss Axio Imager D2 microscope (33). At least 10 microphotographs were taken for every sample under UV/blue emission/excitation channel for DAPI fluorescence (total bacteria), blue/red emission/excitation channel for autofluorescence from Chl-*a* (algae and cyanobacteria), and white light/infrared emission/excitation channel for autofluorescence from BChl-*a* (AAPs). As some part of Chl-*a* autofluorescence is also visible in the infrared (IR) spectrum, only the IR-positive cells that did not show any autofluorescence from Chl-*a* were counted as AAP bacteria.

Fractions of high-nucleic-acid (HNA) bacteria (for normalizing enzymatic activity, see above) were determined using flow cytometry (55). Then, 2-ml water samples were fixed with 2% formaldehyde and stored at 4°C for a maximum of 3 days. Bacterial cells were stained with SYBR Safe (56) and analyzed using an Apogee A50-Micro/NIR (Apogee, Hertfordshire, UK) flow cytometer equipped with 488-nm and 635-nm lasers.

### Bacterial activity.

Bacterial respiration was measured daily over the dark period. Teflon FEP narrow-mouth flasks (Thermo Scientific Nalgene, Waltham, MA) equipped with a sensor spot (SP-PSt3-NAU-D5-YOP), were carefully filled without any bubble formation with 40 ml of prefiltered sample water (<1 μm). The flasks were sealed to avoid gas exchanges and incubated in the dark at 21°C for 24 h. Measurements were carried out by using an optical oxygen sensor (Fibox 3 with Oxyview 6.02 software; Presens GmbH, Regensburg, Germany). The bacterial respiration rates were calculated as the slope of the regression fit of the oxygen concentration versus time (57).

The bacterial biomass production and activity were estimated based on the assimilation rates of radiolabeled leucine and glucose. Tritiated leucine (specific activity, 4,440 GBq mmol^−1^) and glucose (specific activity, 2,220 GBq mmol^−1^; American Radiolabeled Chemicals, St. Louis, MO) were added to a final concentration of 10 nmol liter^−1^, and the samples were incubated for 1 h as described previously by Kirchman et al. (58).

Bacterial biomass production was calculated from the leucine assimilation rates based on conversion factors reported previously (59), and the bacterial growth rate was calculated from bacterial production assuming a cellular carbon content of 10 fg cell^−1^ (60). A ratio of total primary production and bacterial carbon demand was calculated as an indicator of coupling between phytoplankton and bacteria (7). Bacterial carbon demand was calculated as the sum of bacterial respiration and bacterial production (7).

### Bacterial community analysis.

Samples for bacterial community analysis were taken at each sampling point. Between 300 and 400 ml of water was filtered through sterile 0.22-μm Sterivex-GP filter units (Merck Millipore, Darmstadt, Germany). The units were closed, flash-frozen in liquid nitrogen, and stored at –80°C until extraction within a month. Total nucleic acids were extracted as described by Nercessian et al. (61).

The V3-V4 region of bacterial 16S rRNA gene was amplified by using the primers 341F (5′-CCT ACG GGN GGC WGC AG-3′) and 785R (5′-GAC TAC HVG GGT ATC TAA TCC-3′) (62) and sequenced on an Illumina MiSeq (2 × 300 bp) platform at LGC Genomics (Berlin, Germany).

Read quality was evaluated using FastQC v0.11.7 (Babraham Bioinformatics, Cambridge, UK). Primer sequences were trimmed using cutadapt v1.16 (63) and subsequently analyzed in the R/Bioconductor environment using the dada2 v1.6 package (64), as described in the supplemental material. The final ASV table contained from 11,883 to 67,168 reads per sample (25,365 ± 6,210 [mean ± the SD]). Taxonomic assignment was performed using the SILVA 132 database (65), following the Genome Taxonomy Database nomenclature (66). ASVs unclassified at the class level, classified as chloroplasts, or observed less than three times in <20% of the samples were removed. This reduced the number of ASVs by 87% but the total number of reads by only 13%.

### AAP bacterial community analysis.

Samples for AAP community analysis were taken at each sampling point. Its composition was analyzed by amplicon sequencing of the gene encoding the subunit M of the reaction center protein (*pufM*) in bacterial type-2 reaction centers. This gene is the most commonly used marker for diversity studies of AAP bacteria (67).

*pufM* gene amplicons (∼150 bp) were prepared using pufM UniF (5′-GGN AAY YTN TWY TAY AAY CCN TTY CA-3′) and pufM UniR (5′-YCC ATN GTC CAN CKC CAR AA-3′) primers (68) and sequenced on an Illumina MiSeq (2 × 250 bp) platform of the Genomic Service of the Universitat Pompeu Fabra (Barcelona, Spain).

*pufM* reads were processed as described above for 16S rRNA gene amplicons unless specified otherwise (see Text S1 in the supplemental material). The final ASV table contained from 9,808 to 122,584 (58,394.2 ± 14,096.4) reads per sample, except for the sample form the OL treatment, replicate A at 24 h (<2,000 reads), which thus was excluded from the analysis. A manually curated taxonomic database was used for taxonomic assignment. It contained 1,245 unique *pufM* sequences downloaded from the Fungene repository on 16 May 2019 (http://fungene.cme.msu.edu [69]) and 224 *pufM* sequences from metagenomes from the Římov Reservoir (70, 71). ASVs unclassified at the class level and those found less than three times in <20% of the samples were removed, reducing the numbers of ASVs by 67% and the total read number by 12%.

10.1128/mSphere.00354-20.1 TEXT S1 Detailed description of the methods. Download Text S1, PDF file, 0.8 MB. Copyright © 2020 Piwosz et al. 2020 Piwosz et al. This content is distributed under the terms of the Creative Commons Attribution 4.0 International license.

### Statistical analysis.

Differences between the treatments in all measured variables, except for the total bacterial and AAP bacteria communities, were tested for specific time points by using a nonparametric Kruskal-Wallis test and *post hoc* Dunn test with Bonferroni correction of *P* value for multiple comparisons. Changes in the total bacterial and AAP bacteria communities were investigated with distance-based multidimensional methods (PERMANOVA [permutational multivariate analysis of variance], nonmetric multidimensional scaling [nMDS]) that have been shown to allow for reliable ecological interpretation of amplicon data (72). Read numbers were transformed with the variance-stabilizing transformation function of the DESeq2 package (version 1.14.1, blind = FALSE, fitType = “mean”) in R environment (73). RDA was performed to correlate the abundance of heterotrophic bacteria and AAP bacteria, rates of primary production, and specific assimilation rates of leucine and glucose with the concentrations and ratios of POC, PON, and POP. Statistical significance was tested with constrained ordination with a Monte Carlo permutation test (499 permutations). RDA calculation was made with CANOCO, version 5 (74).

### Data availability.

The sequences of 16S amplicons were deposited in the NCBI database under BioSamples SAMN14543044 to SAMN14543088 and those of *pufM* amplicons under BioSamples SAMN14543089 to SAMN14543132 as part of BioProject PRJNA612174.
