# Erratum for Piwosz et al., “Light and Primary Production Shape Bacterial Activity and Community Composition of Aerobic Anoxygenic Phototrophic Bacteria in a Microcosm Experiment”

**DOI:** 10.1128/mSphere.00673-20

**Published:** 2020-07-15

**Authors:** Kasia Piwosz, Ana Vrdoljak, Thijs Frenken, Juan Manuel González-Olalla, Danijela Šantić, R. Michael McKay, Kristian Spilling, Lior Guttman, Petr Znachor, Izabela Mujakić, Lívia Kolesár Fecskeová, Luca Zoccarato, Martina Hanusová, Andrea Pessina, Tom Reich, Hans-Peter Grossart, Michal Koblížek

**Affiliations:** a Center Algatech, Institute of Microbiology, Czech Academy of Sciences, Třeboň, Czechia; b Institute of Oceanography and Fisheries, Split, Croatia; c Department of Aquatic Ecology, Netherlands Institute of Ecology (NIOO-KNAW), Wageningen, The Netherlands; d Great Lakes Institute for Environmental Research, University of Windsor, Windsor, Ontario, Canada; e University Institute of Water Research, University of Granada, Granada, Spain; f Marine Research Centre, Finnish Environment Institute, Helsinki, Finland; g Department of Natural Sciences, University of Agder, Kristiansand, Norway; h Israel Oceanographic and Limnological Research, National Center for Mariculture, Eilat, Israel; i Institute of Hydrobiology, Biology Centre, Czech Academy of Sciences, České Budějovice, Czechia; j Department Experimental Limnology, Leibniz Institute of Freshwater Ecology and Inland Fisheries (IGB), Stechlin, Germany; k Department of Life and Environmental Sciences (DiSVA), Università Politecnica delle Marche, Ancona, Italy; l Haifa University, Haifa, Israel; m Institute of Biochemistry and Biology, Potsdam University, Potsdam, Germany; n Department of Ecosystem Biology, Faculty of Science, University of South Bohemia, České Budějovice, Czechia

## ERRATUM

Volume 5, no. 4, e00354-20, 2020, https://doi.org/10.1128/mSphere.00354-20. Coauthor Izabela Mujakić should be affiliated with an additional institution. Her corrected affiliation information is shown above.

