# Supplementary figures and images for: Light and Primary Production Shape Bacterial Activity and Community Composition of Aerobic Anoxygenic Phototrophic Bacteria in a Microcosm Experiment
Source: mSphere. 2020 Jul 1;5(4):e00354-20. doi: 10.1128/mSphere.00354-20 (PMC7333569; doi:10.1128/mSphere.00354-20)

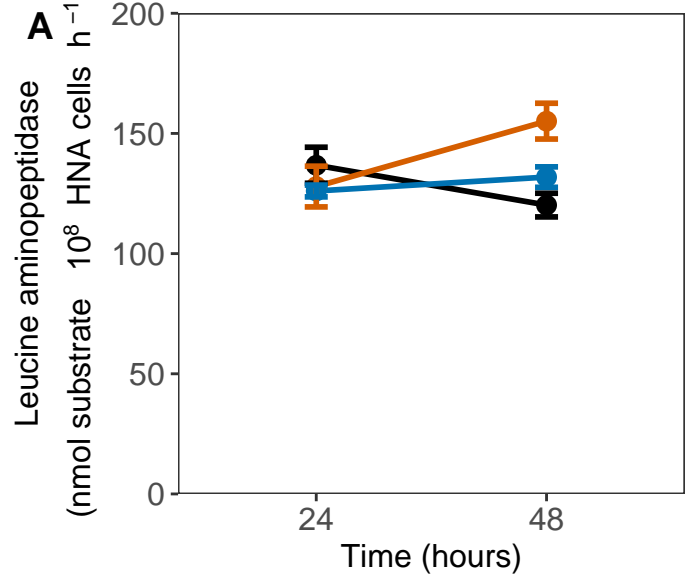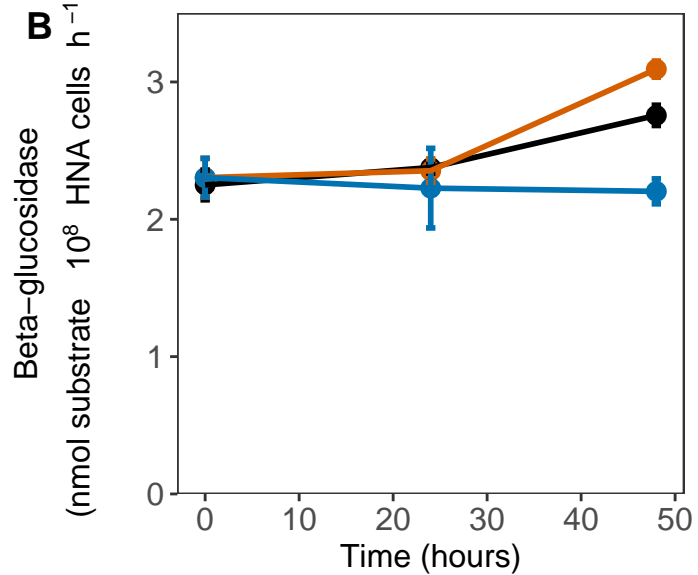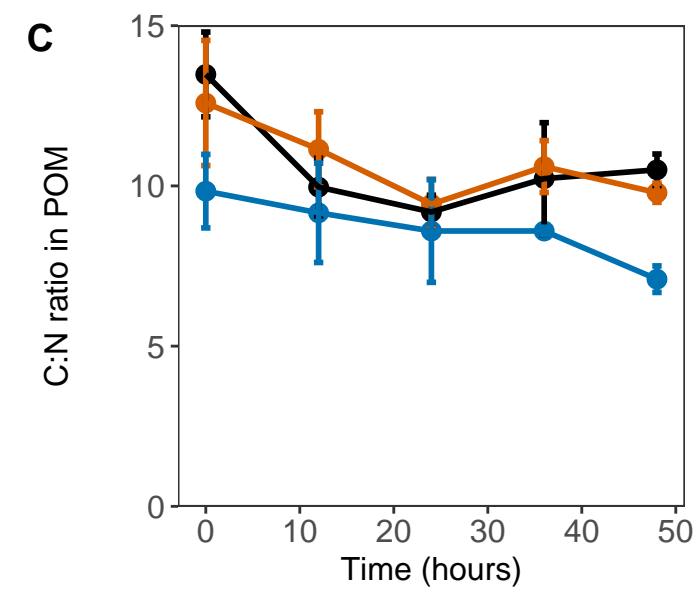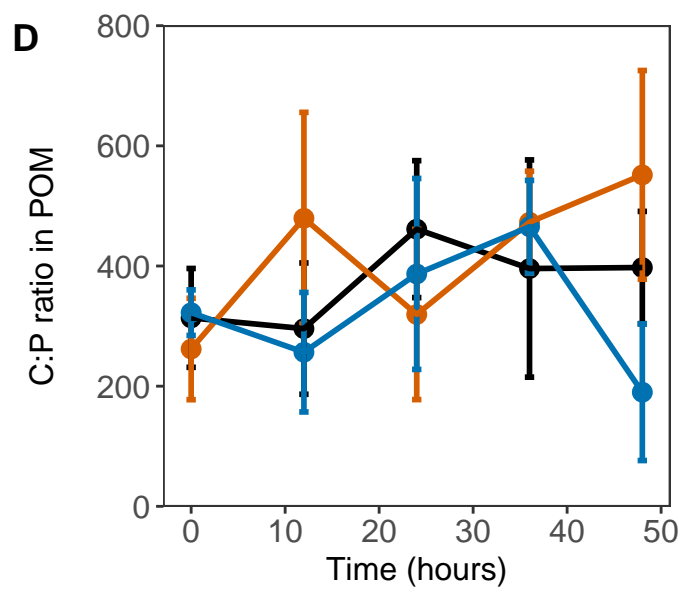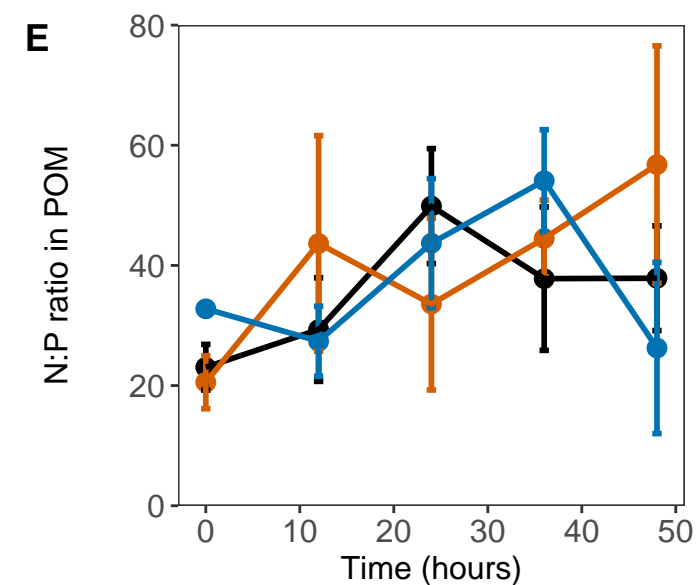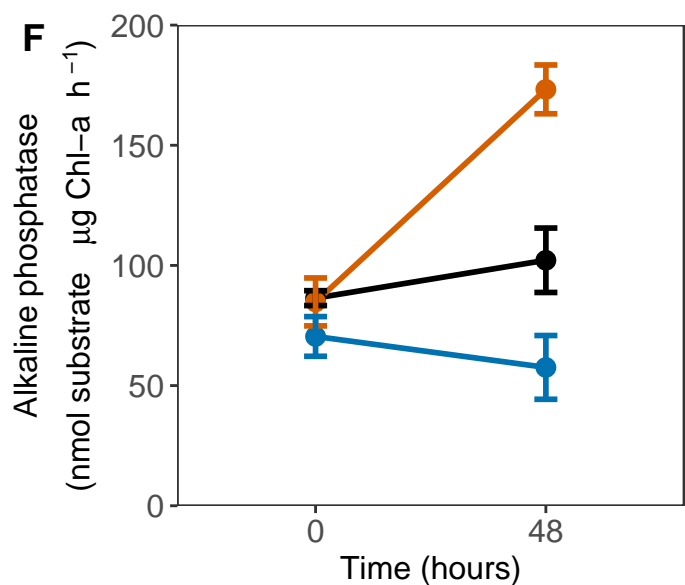

Treatment ■ Low Light ● Optimum Light ▲ Optimum Light + inhibitor

Supplement: FIG S1 [file mSphere.00354-20-sf001.pdf]

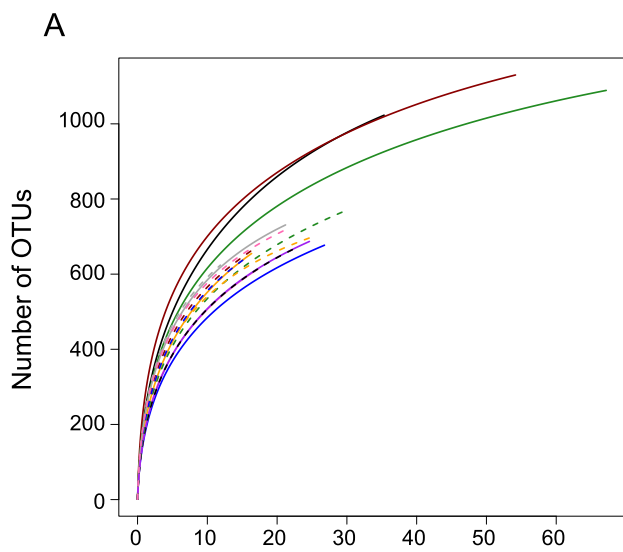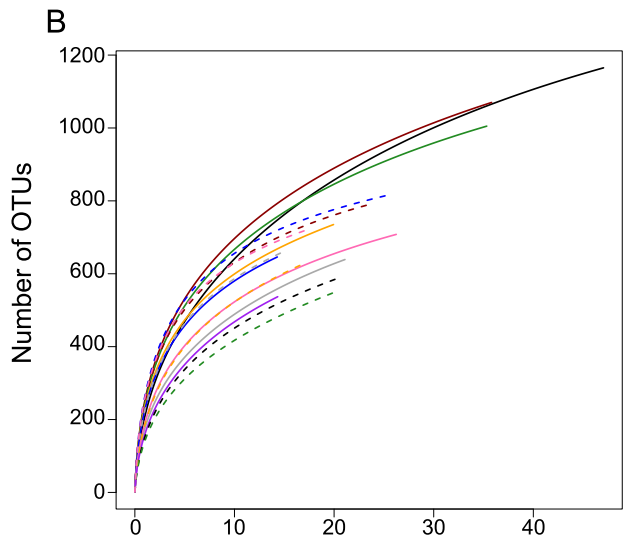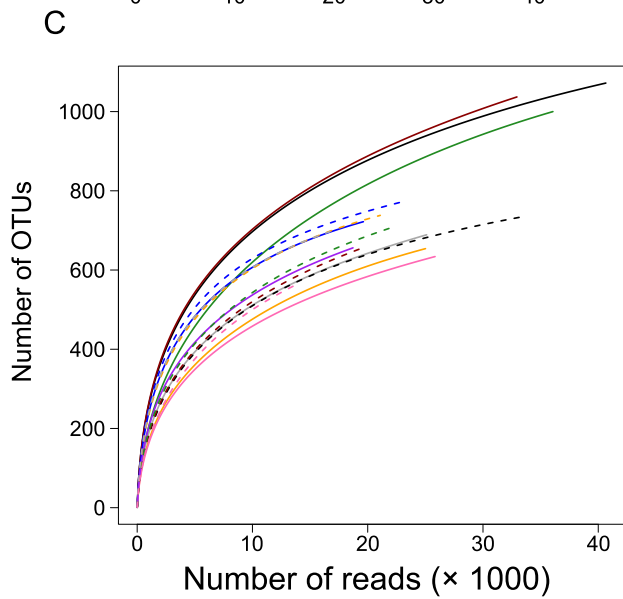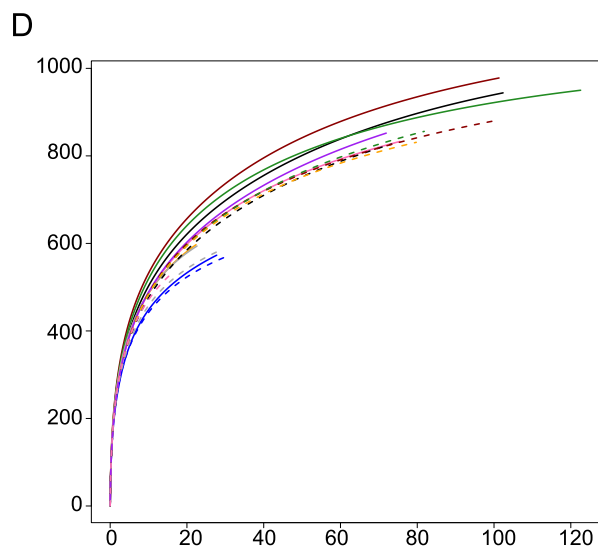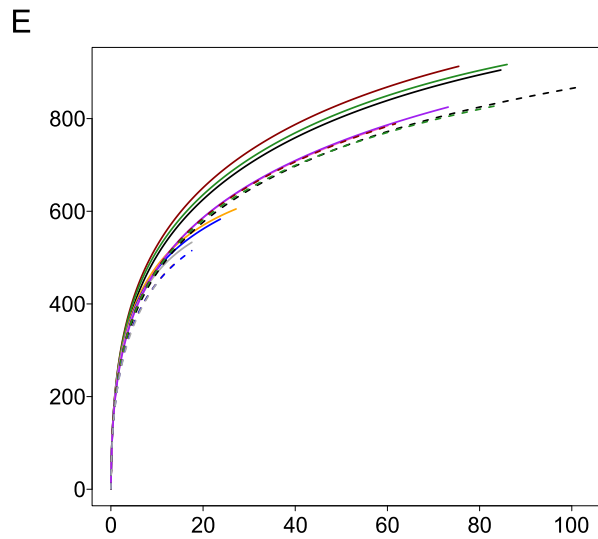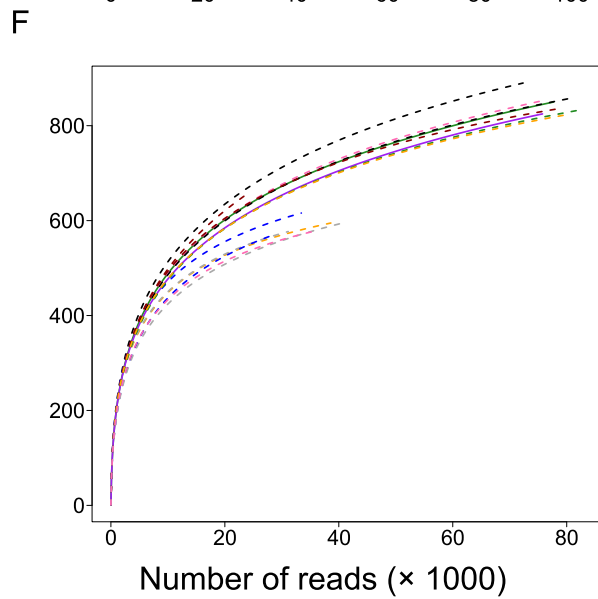

— T0 A    — T12 A    — T24 A    - - T36 A    - - T48 A  
— T0 B    — T12 B    — T24 B    - - T36 B    - - T48 B  
— T0 C    — T12 C    - - T24 C    - - T36 C    - - T48 C

Supplement: FIG S2 [file mSphere.00354-20-sf002.pdf]

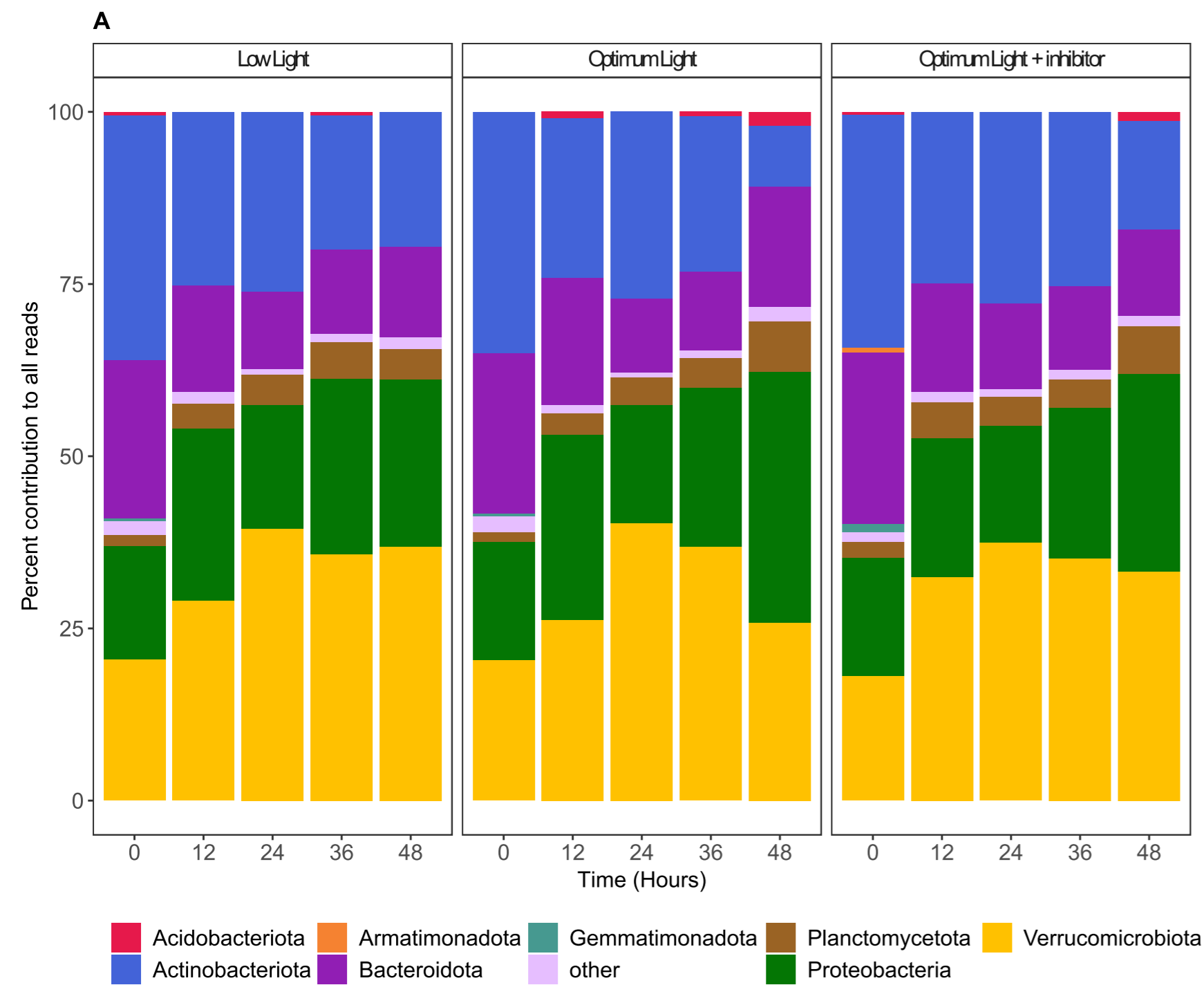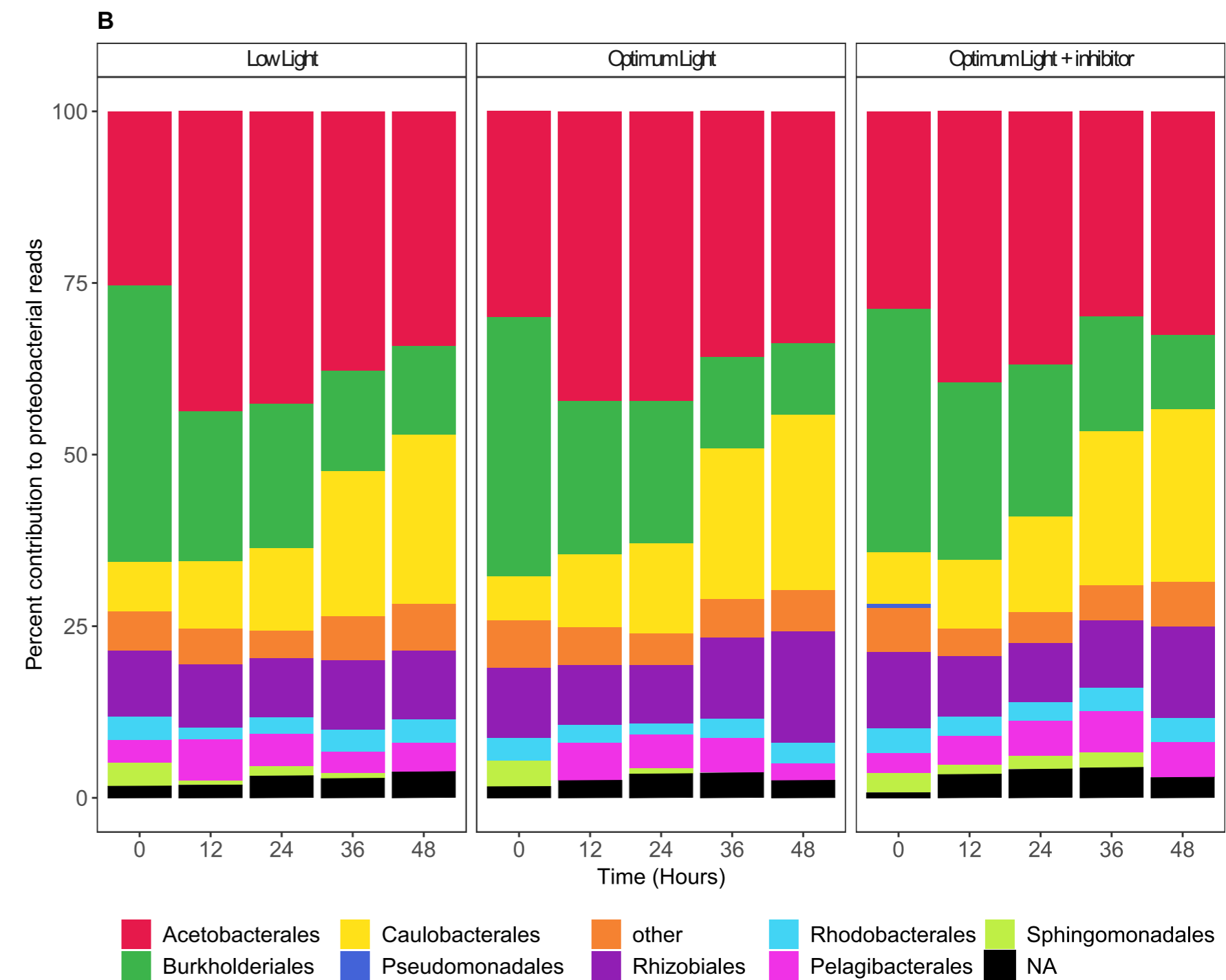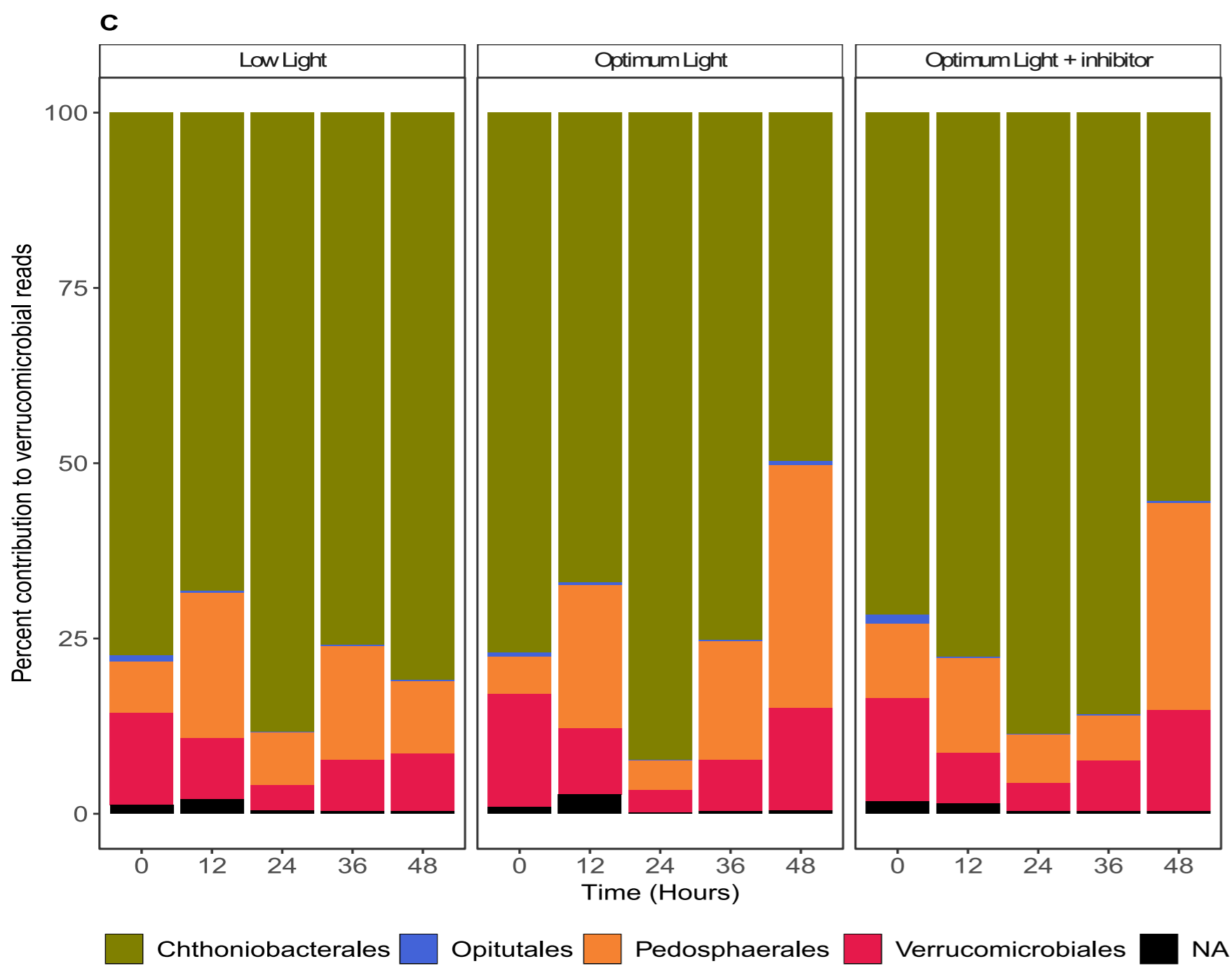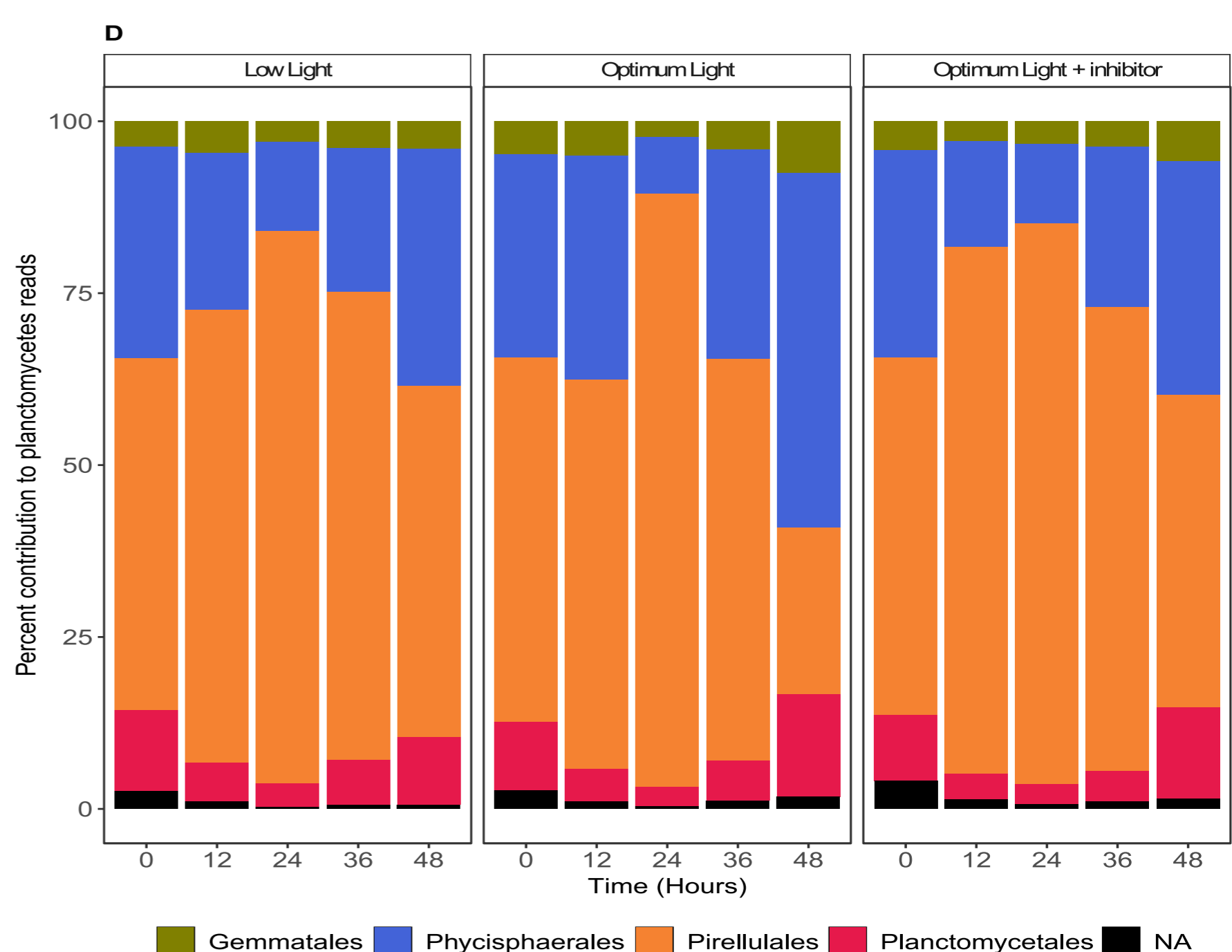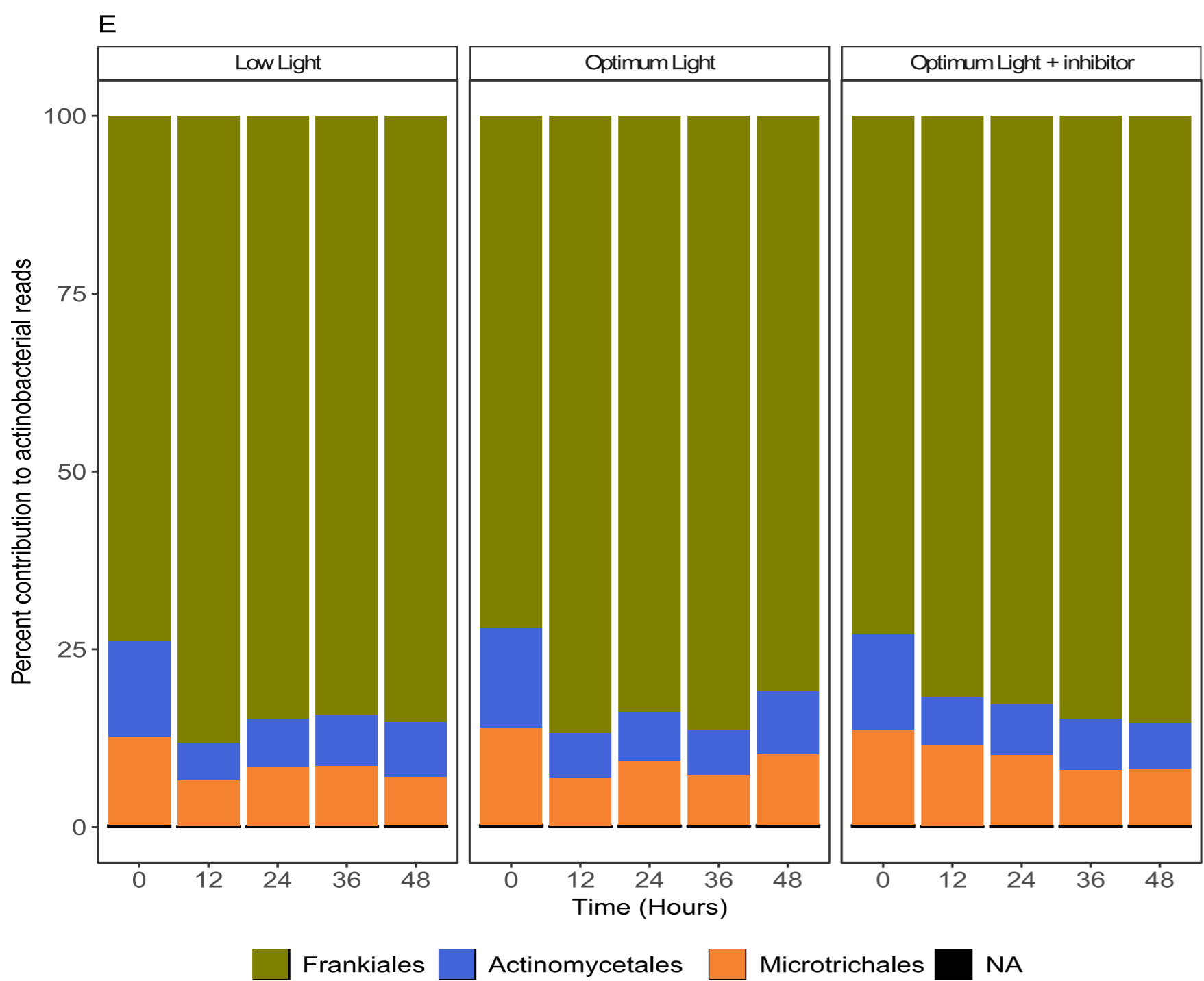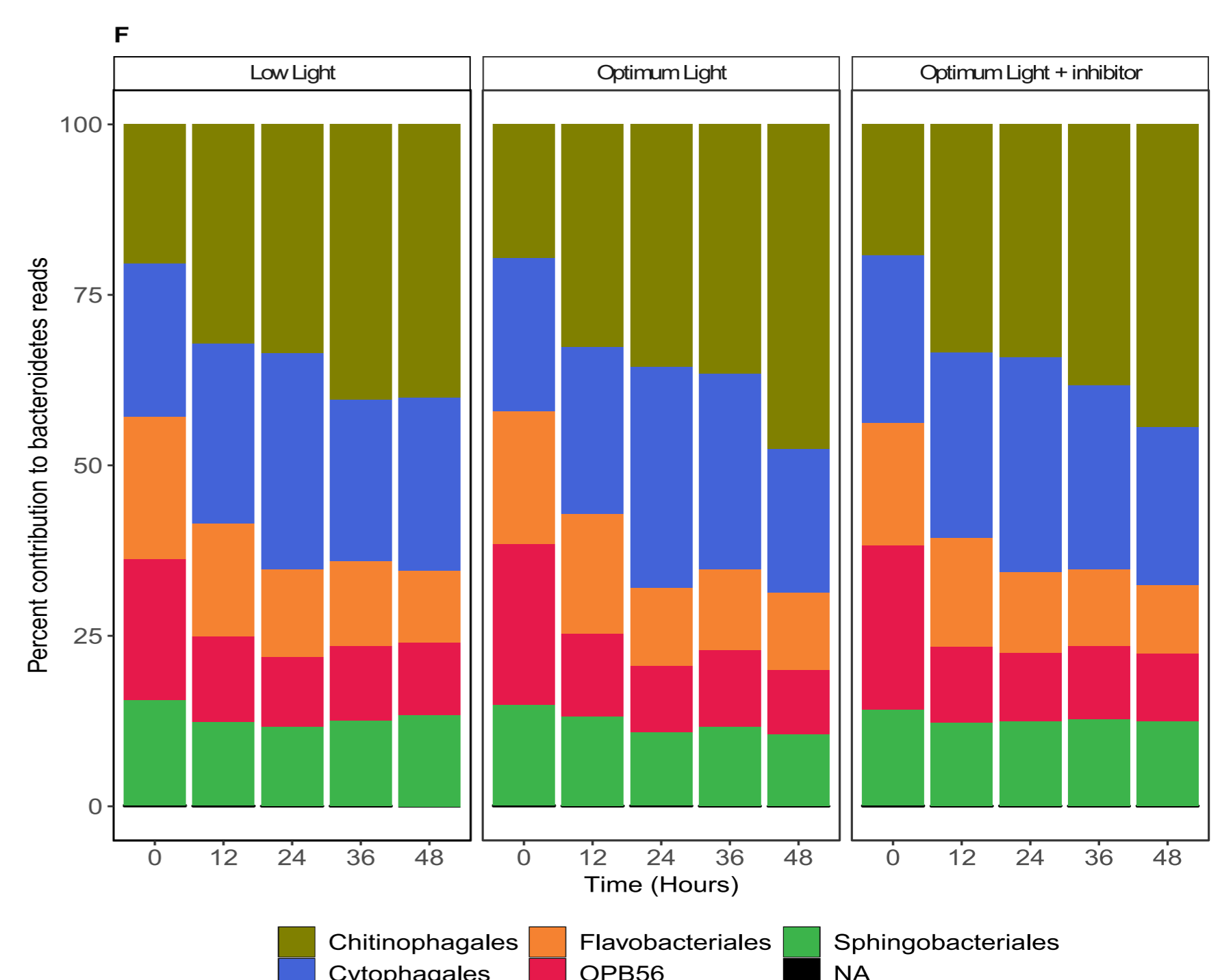

Supplement: FIG S3 [file mSphere.00354-20-sf003.pdf]

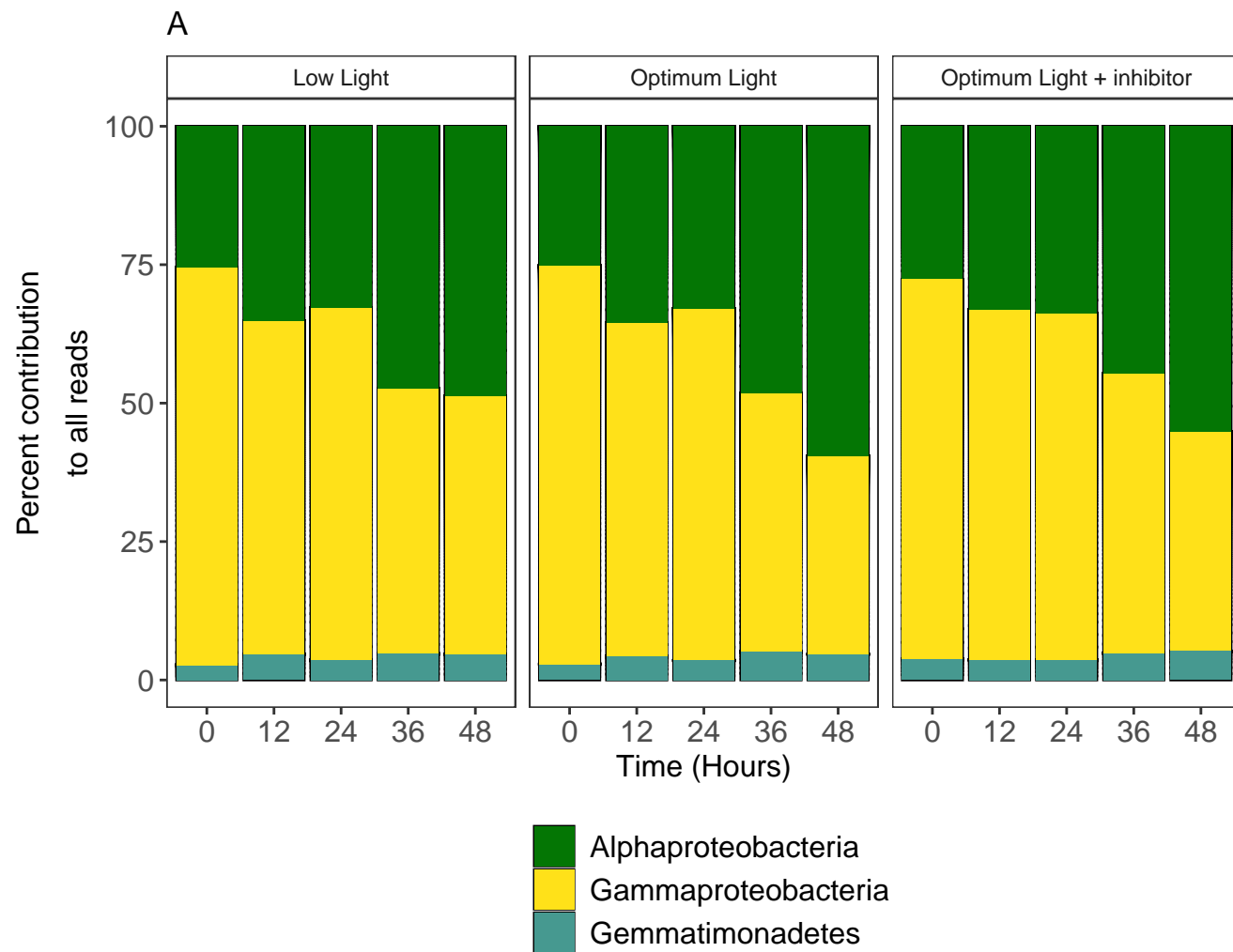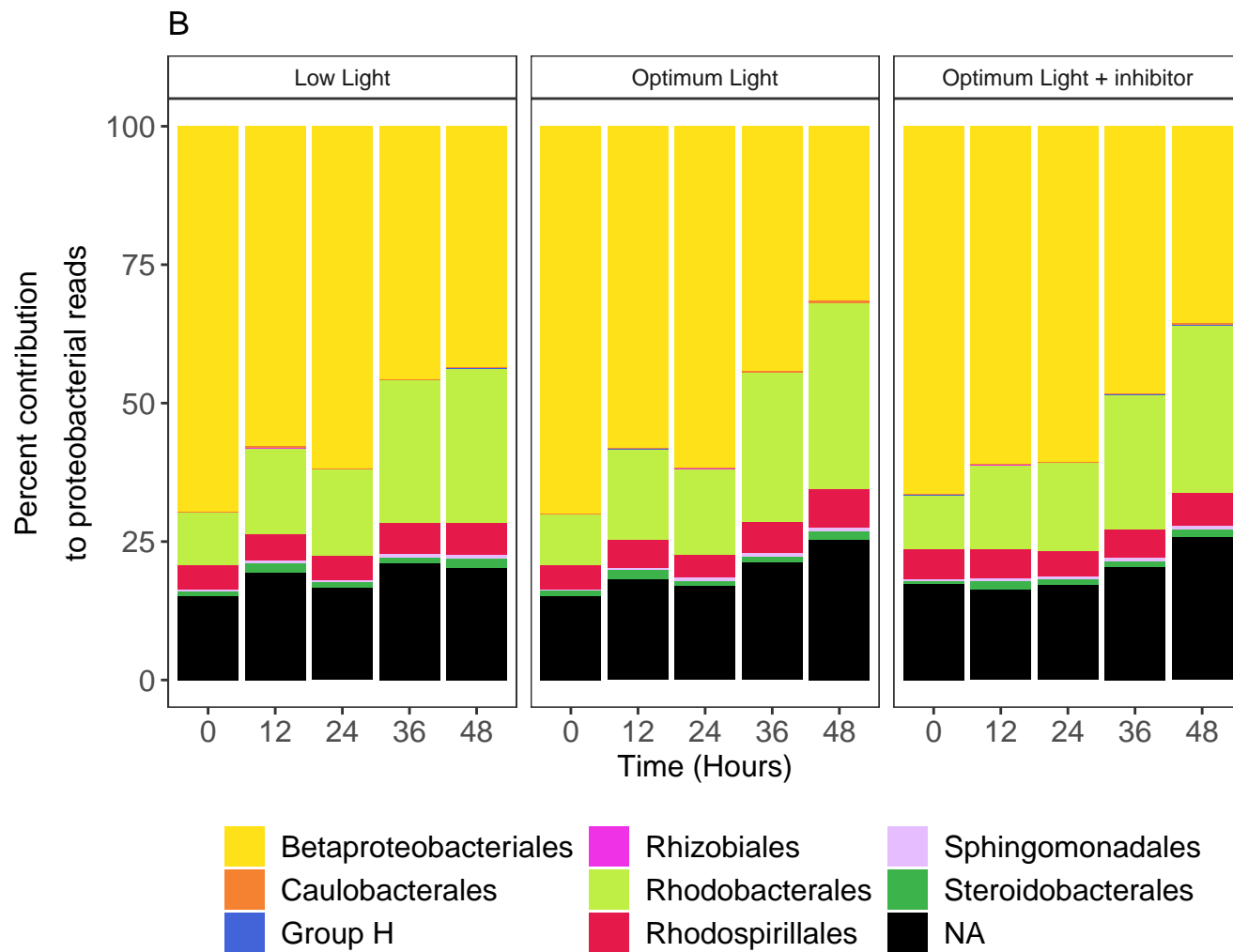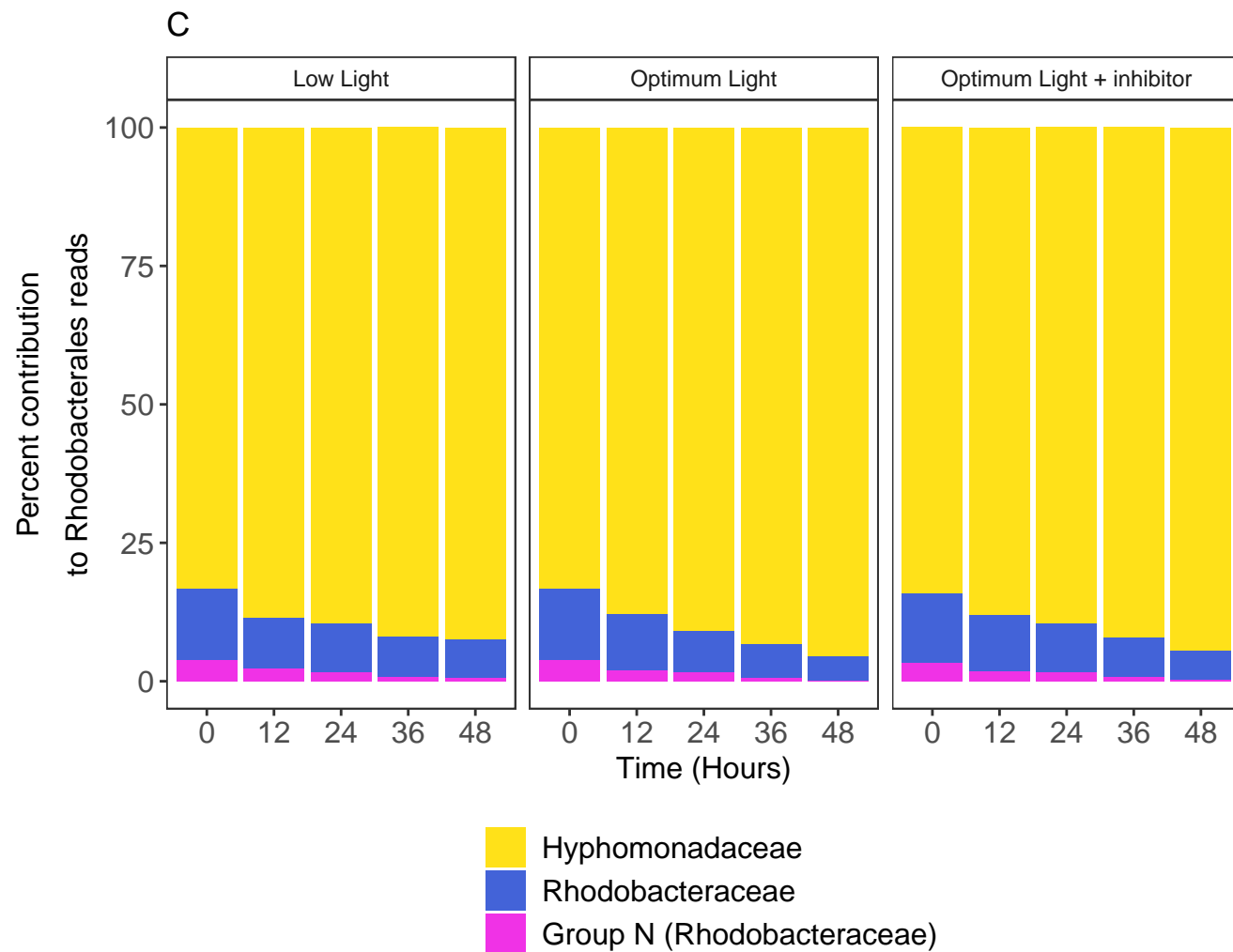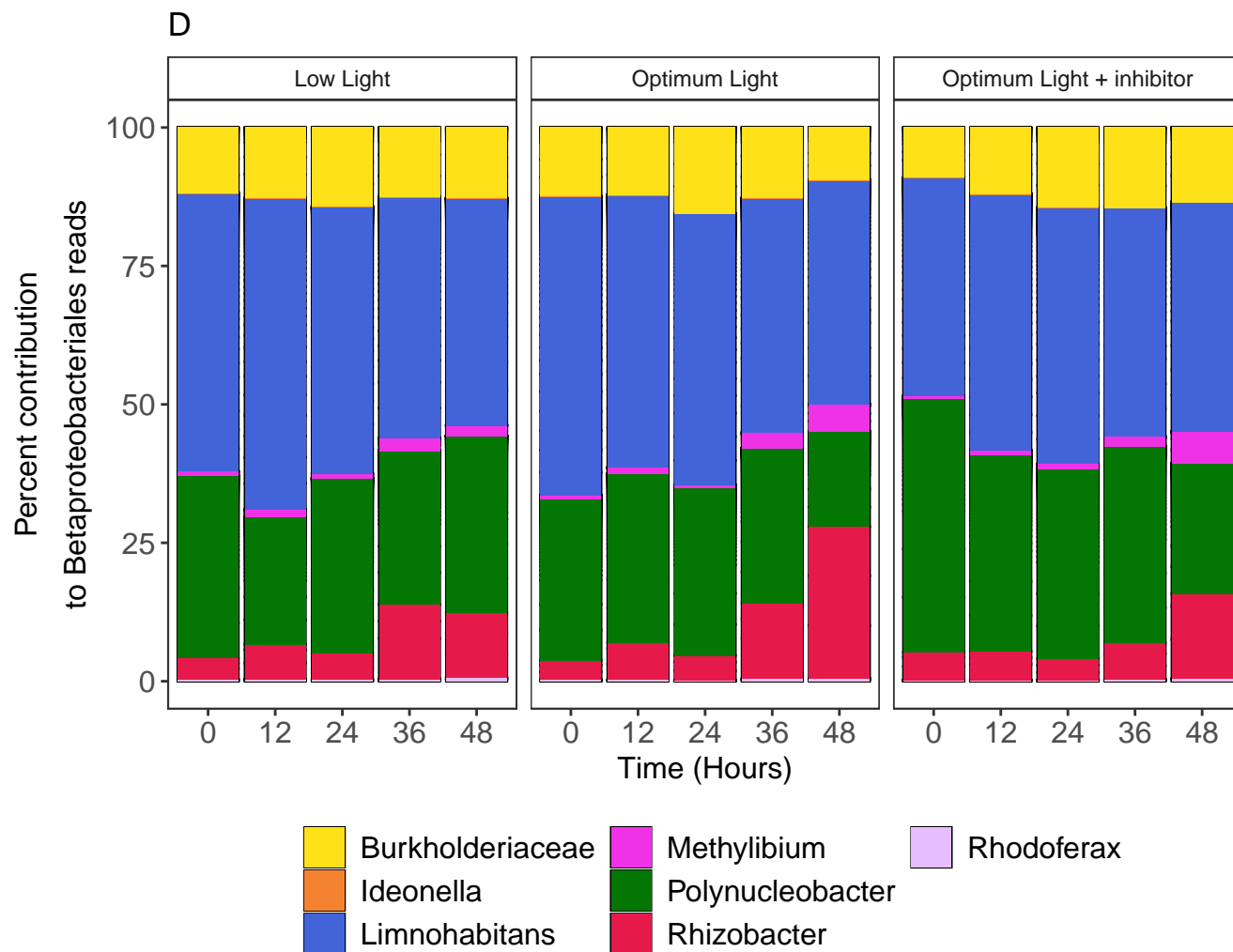

Supplement: FIG S4 [file mSphere.00354-20-sf004.pdf]

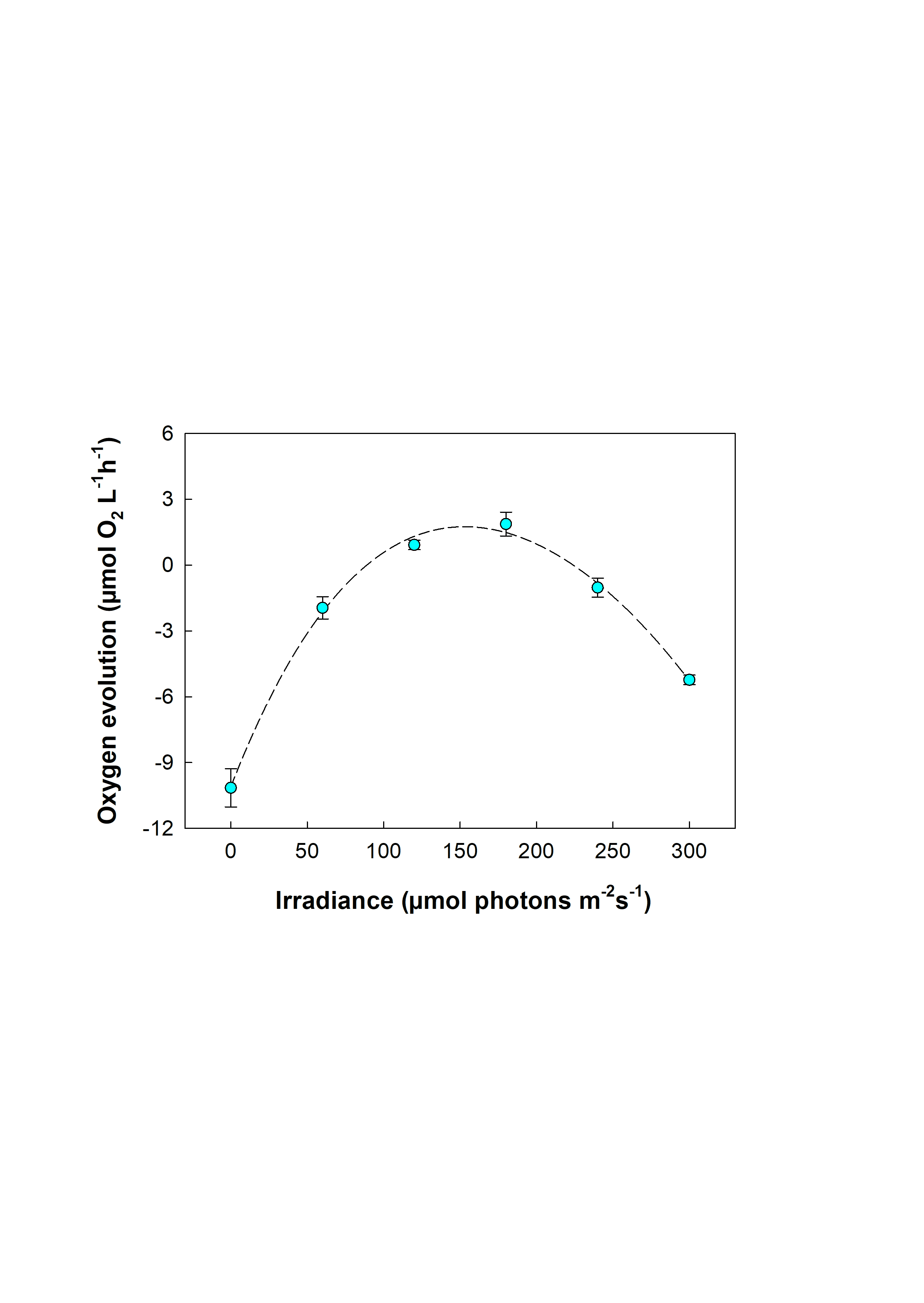

Supplement: FIG S5 [file mSphere.00354-20-sf005.tif]
