# Supplementary material for: Light and Primary Production Shape Bacterial Activity and Community Composition of Aerobic Anoxygenic Phototrophic Bacteria in a Microcosm Experiment
Source: mSphere. 2020 Jul 1;5(4):e00354-20. doi: 10.1128/mSphere.00354-20 (PMC7333569; doi:10.1128/mSphere.00354-20)
Supplement: TABLE S1 [file mSphere.00354-20-st001.pdf]

Supplementary Table S1. Physical, chemical and biological conditions in the Římov Reservoir on August 21, 2017.

Variable	Value
Temperature	21.9°C
pH	9.8
Dissolved organic carbon (DOC)	7.65 mg L^{-1}
Dissolved oxygen	10.6 mg L^{-1}
NO3-N	0.233 mg L^{-1}
NH4-N	23.58 µg L^{-1}
Soluble reactive phosphorous (SRP)	1.7 µg L^{-1}
