# Supplementary material for: Light and Primary Production Shape Bacterial Activity and Community Composition of Aerobic Anoxygenic Phototrophic Bacteria in a Microcosm Experiment
Source: mSphere. 2020 Jul 1;5(4):e00354-20. doi: 10.1128/mSphere.00354-20 (PMC7333569; doi:10.1128/mSphere.00354-20)
Supplement: TEXT S1 [file mSphere.00354-20-s0001.pdf]

Light and primary production shape bacterial activity and community composition of aerobic anoxygenic phototrophic bacteria in a microcosm experiment.

Kasia Piwosz [a]#, Ana Vrdoljak[b], Thijs Frenken[c,d], Juan Manuel González-Olalla[e], Danijela Šantić[b], R. Michael McKay[d], Kristian Spilling[f,g], Lior Guttman[h], Petr Znachor[i], Izabela Mujakić[a], Lívia Kolesár Fecskeová[a], Luca Zoccarato[j], Martina Hanusová[a], Andrea Pessina[k], Tom Reich[l], Hans-Peter Grossart[j,m]#, Michal Koblížek[a]

[a]Center Algatech, Institute of Microbiology Czech Acad Sci, Třeboň, Czechia

[b]Institute of Oceanography and Fisheries, Split, Croatia

[c]Department of Aquatic Ecology, Netherlands Institute of Ecology (NIOO-KNAW), Wageningen, The Netherlands

[d]Great Lakes Institute for Environmental Research, University of Windsor, Windsor, Ontario, Canada

[e]University Institute of Water Research, University of Granada, Granada, Spain

[f]Marine Research Centre, Finnish Environment Institute, Helsinki, Finland

[g]Department of Natural Sciences, University of Agder, Kristiansand, Norway

[h]Israel Oceanographic and Limnological Research, National Center for Mariculture, Eilat, Israel.

[i]Institute of Hydrobiology, Biology Centre Czech Acad Sci, České Budějovice, Czechia

[j]Department Experimental Limnology, Leibniz Institute of Freshwater Ecology and Inland Fisheries (IGB), Stechlin, Germany

[k]Department of Life and Environmental Sciences (DiSVA), Università Politecnica delle Marche, Ancona, Italy

[l]Haifa University, Haifa, Israel

[m]Institute of Biochemistry and Biology, Potsdam University, Potsdam, Germany

# Address correspondence to Kasia Piwosz: piwosz@alga.cz; Hans-Peter Grossart: hgrossart@igb-berlin.de

Contents

Setting up the experiment 3

Nutrients concentrations 3

Primary production 4

Concentrations of pigments 5

Extracellular enzymatic activity 6

Carbon, nitrogen and phosphorous 7

Bacterial activity 8

Bacterial community analysis 10

AAP bacterial community analysis 11

Statistical analysis 12

References 13

Setting up the experiment

Water was collected from 0.5 m depth near the dam of the Římov Reservoir (48.846^{o}N 14.487^{o}E) on August 21, 2017 in the afternoon using a Friedinger sampler (1). Ambient temperature, pH, and oxygen concentration were measured with a YSI EXO2 multiprobe. Samples for dissolved nutrients were immediately transported in a cooler to the laboratory and proceed as describe below.

The water collected for the experiment was pre-filtered through a 200 µm mesh to remove zooplankton. Aliquots of 8L were distributed into nine clean polyethylene terephthalate (PET) bottles using Tygon tubing (Saint-Gobain, Courbevoie, France) and a peristaltic pump. The incubation was performed at the ambient water temperature (22°C), over a 12:12 h dark:light period. White light was provided by banks of Osram Dulux L 55W/865 (Osram, Munich, Germany) luminescent tubes with a spectral temperature of 6500 K. Sampling started after 12 h of the dark incubation, before the onset of the first light period (T0), and was conducted every 12 h (before the onset and at the end of the light periods) for the following 48 h.

Nutrients concentrations

Samples for dissolved nutrients were immediately transported in a cooler to the laboratory where they were filtered through glass fiber filters with 0.4 µm nominal porosity (GF-5, Macherey-Nagel, Düren, Germany). Concentrations of soluble reactive phosphorus (SRP) and total phosphorus (TP) were determined spectrophotometrically (2, 3). Concentrations of nitrate and ammonium were determined following the protocols of L. Procházková (4) and J. Kopáčkek and L. Procházková (5).

Dissolved organic carbon (DOC), total inorganic nitrogen (TIN) and dissolved organic nitrogen (DON) were measured with a TOC 5000A analyzer (Shimadzu, Kyoto, Japan).

Primary production

Primary production was measured using a slight modification of the ^{14}C radiolabel method (6) Total activity of 1.85 kBq $H^{14}CO_3$ was added to 1 mL of a sample placed in a 5-ml scintillation vial. Three sets of incubations were prepared for measuring (i) total carbon fixation, (ii) the fraction of primary production released as dissolved organic carbon (DOC), and (iii) dark CO_2 assimilation. All vials were incubated next to the experimental units (same light and temperature) for 2 h in technical duplicates, and the vials for the dark measurements were completely covered with aluminium foil. After the incubations, 100 µl of 1 mol L^{-1} HCl were added to the vials to volatilize unincorporated $H^{14}CO_3$. For determining the fraction of primary production released as DOC, the total volume was gently filtered through a 0.2 µm polycarbonate filter into a clean scintillation vial, and the acid was subsequently added. The vials were left for 24 h in an exhaust hood before 4 mL of scintillation liquid (Perkin Elmer, Waltham, MA, USA) were added. The activity was determined in a scintillation counter (Perkin Elmer). The primary production was calculated knowing the added ^{14}C concentration, the dissolved inorganic carbon (DIC) pool (see below) and the fixed fraction of the radiolabeled carbon according to **Equation 1**:

$$CFIX = \frac{DMP \times DIC \times 1.05}{TOT \times t}$$

where:

CFIX the carbon fixation rate (µmol C L^{-1} h^{-1})

DPM: disintegrations per minute in samples, measured with the scintillation counter,

DIC: the total dissolved inorganic carbon pool

TOT: the total activity added (DMP)

t: incubation time (h)

The factor 1.05 is added to account for $^{14}CO_2$ being taken up slower than $^{12}CO_2$ (7)

The pH of the sample was measured with a pH meter (Inolab pH 720, WTW Xylem Inc. Rye Brook, NY, US). Alkalinity was determined by an automatic titrator Metrohm 877 (Metrohm, Herisau, Switzerland) where 0.1 µmol L^{-1} HCl was added and the pH continuously measured. The dissolved inorganic carbon (DIC) concentration was calculated knowing the sample pH and alkalinity. Carbon fixation was determined at the beginning and at the end of the light period, and for the accumulated carbon fixation, the values measured for these two periods were used for the time these measurements took place. The development of carbon fixation for the rest of the day was estimated by linear regression, and the measured/estimated carbon fixation was added each hour to yield the accumulated carbon fixation assuming no carbon was fixed during the dark period.

Concentrations of pigments

Phytoplankton biomass from 0.6-0.75 L (LL and OL treatments) or of 0.9-1 L (OL-Inh) was collected by filtration. Pigments were extracted from homogenized filters (15 mL glass homogenizer and power-driven Teflon piston) in 8 mL of 7:2 v/v acetone:methanol mixture. Clear extracts were analyzed using a Prominence-i HPLC system (Shimadzu Inc., Japan) equipped with a UV-VIS diode-array detector. Pigments were separated using a heated (40°C) Phenomenex Luna 3µC8(2) 100 Å

column with binary solvent system A: 20% 28 mM ammonium acetate + 80% methanol, B: 100% methanol, with flow rate of 0.8 mL min^{-1}. The peak for Chl a was registered at 656 nm and the pigment concentration in the original sample was calculated from the peak area. The HPLC system was calibrated using 100% methanol extracts of *Synechocystis* sp. PCC6803 and *Rhodobacter sphaeroides* with known concentrations of Chla or BChl a, respectively.

Extracellular enzymatic activity

Extracellular enzyme activities corresponding to alkaline phosphatase (APase; E.C. 3.1.3.1), β-1,4-glucosidase (βGase; E.C. 3.2.1.21) and leucine aminopeptidase (LAPase; E.C. 3.4.11.1) were measured in following general protocols conveyed in Hoppe (1993). Whole water was dispersed to triplicate methacrylate cuvettes (2.5 ml) and incubated with 100 µmol L^{-1} of fluorogenic substrate; 4-methylumbelliferyl phosphate, 4-methylumbelliferyl β-D-glucopyranoside and L-leucine-7-amido-4-methylcoumarin (Sigma-Aldrich, St. Louis, MO, USA), respectively. Sodium bicarbonate (4 mmol L-1) was substituted for whole water in substrate controls, whereas quench standards were prepared using whole water and 2 µmol L-1 of reference standards 4-methylumbelliferone (APase, βGase) or 7-amino-4-methylcoumarin (LAPase) (Sigma-Aldrich). Enzyme-catalyzed fluorescence was determined within 1 h of substrate incubation using an AMINCO Bowman Series 2 Luminescence Spectrophotometer (Spectronic Unicam, Cambridge, UK). Monochromators were set for appropriate excitation and emission spectra of the fluorochromes produced in each assay (364 and 445 ± 16 nm, respectively, for methylumbelliferyl substrates; and 380 and 440 ± 16 nm for the methylcoumarin substrate). Enzyme activities were calculated using reference standards prepared in 4 mmol L^{-1} sodium bicarbonate.

The sensitivity of detection of the fluorescent products of methylumbelliferyl substrates is pH-dependent with maximum fluorescence obtained at pH 10.3 (8). A water column physico-chemical profile made at the Římov reservoir at the time of sample collection showed epilimnetic (upper 2 m layer) pH to be 9.8, a condition supporting maximum fluorescence. Likewise, values of quench standards did not vary from reference standards prepared in sodium bicarbonate at any point during the manipulation experiment indicating that sample pH did not affect fluorescence measured in the assays.

Carbon, nitrogen and phosphorous

Particulate organic carbon (C), nitrogen (N) and phosphorus (P) were determined by collecting 60 mL of seston on a prewashed GF/F filter (Whatman, Maidstone, UK) using a syringe and a syringe filter holder (25 mm MilliporeSigma Swinnex® Syrenge Filer Holder, Merck KGaA, Darmstadt, Germany). Blank filters were also measured.

Afterwards, filters were dried overnight at 60°C and stored in a desiccator in the dark. For C and N analyses, a subsample (18%) of every filter was taken by a hole puncher, folded into a tin cup and analyzed on a FLASH 2000 organic elemental analyzer (Brechbueler Incorporated, Interscience B.V., Breda, The Netherlands).

Particulate organic P was analyzed (Eaton, 2005) by first combusting the remainder of the filter (82%) for 30 min at 550°C in Pyrex glass tubes, followed by a digestion step with 3 mL persulfate (2.5%) for 30 min at 120°C. This digested solution was measured for PO_4^{3-} on a QuAAtro39 AutoAnalyzer (SEAL Analytical Ltd, Southampton, UK) following Armstrong et al. (1967).

Bacterial activity

Samples and killed controls were spiked with tritiated monomers (American Radiolabeled Chemicals, St. Louis, MO, USA) to a final concentration of 10 nmol L^{-1}. Sterile formalin was added to the killed controls to a final concentration of 1.5%. Samples were incubated for 1 h at the same temperature and light conditions as the corresponding experimental units. The incubations were terminated with formalin, as for the killed controls. Samples were then filtered onto nitrate cellulose filters (pore size 0.2 µm, diameter 25 mm, Pragopor, Prague, Czech Republic) and processed following the protocol of D. Kirchman et al. (9). Filter were washed twice with 2.5 mL of ice-cold 5%, and then twice with 2.5 mL of ice-cold 80% ethanol. Filters were placed in the scintillation vials (Fisher) and air-dried overnight. Dried filters were dissolved in 1 ml of ethyl acetate. Then, 5 ml of Ultima Golt LLT scintillation cocktail (PerkinElmer) was added and samples were gently mixed and left in the dark for 48 h.The radioactivity in the samples was measured using on a Tri-Carb 2810 TR scintillation counter (PerkinElmer).

Equations:

Equation 2: Assimilation rates of leucine/glucose:

$$AR = \frac{DMP \times CF}{SA \times t \times V}$$

where:

AR: assimilation rate (mmol L^{-1} h^{-1})

DPM: disintegrations per minute measured with the scintillation counter (mean of triplicates-killed controls)

CF: conversion factor DPM to Ci: 4.5×10^{-13}

SA: specific activity (Ci $mmol^{-1}$)

t: incubation time (h)

V: Filtered sample volume (L)

Equation 3: Specific assimilation rates of leucine/glucose:

$$SAR = \frac{AR}{BA}$$

where:

SAR: specific assimilation rate (mmol $cell^{-1}$ h^{-1})

AR: assimilation rate (mmol L^{-1} h^{-1}; see equation 1)

BA: bacterial abundance (cells L^{-1})

Equation 4: Bacterial biomass production. Conservative value of 1 for isotope dilution has been assumed (10).

$$BP = \frac{AR \times MLeu}{FLeu \times CC}$$

where:

BP: Bacterial production (gC L^{-1} h^{-1})

AR: assimilation rate (mmol L^{-1} h^{-1}; see equation 1)

MLeu: molecular weight of leucine

FLeu: Fraction of leucine in proteins (7.3%; (11))

CC: Cellular carbon per protein (0.86, (11))

Equation 5: Bacterial growth rate:

$$\mu = \frac{BP}{BA \times CB}$$

where:

BP: Bacterial production (gC L^{-1} h^{-1}; see equation 3)

BA: bacterial abundance (cells L^{-1})

CB: Carbon per cell conversion factor (10 fgC $cell^{-1}$ (12))

Bacterial community analysis

16S rRNA gene amplicons were prepared using the primers set 341F-785R for the V3-V4 region (13). PCR was performed in a 50 µL reaction, using MyTaq Red DNA Polymerase (Bioline, Germany) with the following reaction conditions: 94°C for 2 min, 32 cycles at 94°C for 15 s, 53°C for 15 s, 72°C for 30 s, and a final extension at 72°C for 5 min.

The number of reads per sample ranged from 18,877 to 143,437 (average ± standard deviation: 47,495.8±11,132.9). The quality of reads in each sample was evaluated using FastQC v0.11.7. The primers' sequences were trimmed using cutadapt v1.16 (14), and subsequent analyses were done in the R/Bioconductor environment using the dada2 v1.6 package (15). Low quality sequences were filtered out with the filterAndTrim function (truncLen=c(250, 210) maxN=0 (no N allowed), maxEE=c(2, 2), truncQ=2, rm.phix = TRUE). After the quality filtering and denoising, the number of reads per sample ranged from 12,512 to 103,404 (32,171.1±16,062.9). After error learning and sequence variants interference, the sequences were merged, and amplicon sequence variant

(ASV) table was produced. Chimeric sequences were removed using the consensus method of the removeBimeraDenovo function. The final ASV table contained from 11,883 to 67,168 reads per sample (25,365.3± 6,209.7).

AAP bacterial community analysis

*Puf*M amplicons were prepared using pufM UniF and pufM UniR primers (16) and Phusion HotStart II High Fidelity PCR MasterMix (Thermo Scientific). PCR conditions were the following: initial denaturation for 3 min at 98°C, 33 cycles of 98°C for 10 s, 52 °C for 10 s, 72°C for 30 s, final elongation at 72°C for 5 min. The amplicons were purified from the gel using the Wizzard SV Gel and PCR clean system (Promega) and quantified with Qubit dsDNA HS assay. Library preparation (2x250 bp) and sequencing on a MiSeq Illumina was performed by Genomic Service of the Universitat Pompeu Fabra (Barcelona, Spain). The number of reads per sample ranged from 10,699 to 136,285 (average ± standard deviation: 65,584.8±15,288.3), except for the sample form OL treatment replicate A time 24 hrs (< 2,000 reads), which was excluded from further analysis.

The quality of reads in each sample was evaluated using FastQC v0.11.7. The primers' sequences were trimmed using cutadapt v1.16 (14), and subsequent analyses were done in the R/Bioconductor environment using the dada2 v1.6 package (15). Forward and reverse reads were truncated to 160 bp and trimmed by 40 bp at the 5' end. Low quality sequences were filtered out with the filterAndTrim function (truncLen=c(160, 160), trimLeft=c(40.40), maxN=0 (no N allowed), maxEE=c(2, 2), truncQ=2, rm.phix = TRUE). The number of reads per sample ranged from 10,089 to 130,819 (62,524.8±14,894.5) upon the quality filtering, and from 9,808 to 122,584 (58,394.2±14,096.4) after the chimera removal using the consensus method of the removeBimeraDenovo function.

Statistical analysis

The differences between the treatments in all measured variables except for bacterial and AAP communities were tested for specific time points using non-parametric Kruskal-Wallis test and post-hoc Dunn test with Bonferroni correction of p-value for multiple comparisons. Calculations were done in R environment using package dunn.test version 1.3.5 (17).

Changes in total bacterial and AAP communities were investigated with distance based multidimensional methods that have been show to allow for reliable ecological interpretation of amplicon data (Piwosz et al. in press). Differences between the treatments were calculated with Permutational MANOVA (PERMANOVA) with partial sums of squares type III and Monte Carlo pair-wise tests in Primer (version 7.0.13) with PERMANOVA+ 1 add on (e-Primer, Plymouth, UK). Read numbers were transformed with the varianceStabilizingTransformation function of the DESeq2 package (version 1.14.1, blind=FALSE, fitType = "mean") in R environment (18). nMDS plots were calculated from Bray-Curtis distance matrixes using the phyloseq package (version1.19.1) (19). Figures were done using the ggplot2 package (version 3.2.0) (20), the gridExtra package (version 2.3) (21), cowplot package (version 1.0.0) and pheatmap package (version 1.0.12).

The difference in relative abundance of specific ASVs between the OL (control) versus OL-Inh and LL treatments was tested using DESeq function of the DESeq2 package (18) at significance level alpha<0.01 for Benjamini-Hochberg adjusted p-value.

Redundancy analysis (RDA) was used to extract and summarize the variation in a set of response variables that can be explained by a set of environmental (explanatory) variables (Van den Brink et al., 2003; Zuur et al., 2007; Paliy and Shankar, 2016). The null hypothesis that the response

variables (heterotrophic bacteria, AAPs, primary and bacterial production, glucose and leucine uptake) were independent of the explanatory variables (concentrations of carbon, nitrogen and phosphorus) was tested using constrained ordination with a Monte Carlo permutation test (499 permutations). RDA calculation was made with CANOCO, version 5 (Ter Braak and Šmilauer, 2012) using untransformed data.

References

1. Znachor P, Nedoma J, Hejzlar J, Seda J, Kopacek J, Boukal D, Mrkvicka T. 2018. Multiple long-term trends and trend reversals dominate environmental conditions in a man-made freshwater reservoir. Sci Total Environ 624:24-33.

2. Murphy J, Riley JP. 1962. A modified single solution method for the determination of phosphate in natural waters. Analytica Chimica Acta 27:31-36.

3. Kopáček J, Hejzlar J. 1993. Semi-Micro Determination of Total Phosphorus in Fresh Waters with Perchloric Acid Digestion. International Journal of Environmental Analytical Chemistry 53:173-183.

4. Procházková L. 1959. Bestimmung der Nitrate im Wasser. Fresenius' Zeitschrift für analytische Chemie 167:254-260.

5. Kopáčkek J, Procházková L. 1993. Semi-Micro Determination of Ammonia in Water by the Rubazoic Acid Method. International Journal of Environmental Analytical Chemistry 53:243-248.

6. E. S-N. 1952. The use of radioactive carbon (14C) for measuring organic production in the sea. J Cons Int Explor Mer 18:117–140.

7. Peterson BJ. 1980. Aquatic Primary Productivity and the ^{14}C-CO_2 Method: A History of the Productivity Problem. Ann Rev Ecol Syst 11:359-385.

8. Hoppe H-G. 1993. Use of fluorogenic model substrates for extracellular enzyme activity (EEA) measurement of bacteria. Lewis Publishers, Boca Raton, FL.

9. Kirchman D, Knees E, Hodson R. 1985. Leucine incorporation and its potential as a measure of protein-synthesis by bacteria in natural aquatic system. Applied and Environmental Microbiology 49:599-607.

10. Kirchman D. 2001. Measuring bacterial biomass production and growth rates from leucine incorporation in natural aquatic environments. Methods in Microbiology - METH MICROBIOL 30:227-237.

11. Simon M, Azam F. 1989. Protein content and protein synthesis rates of planktonic marine bacteria Marine Ecology Progress Series 51:201-213.

12. Fukuda R, Ogawa H, Nagata T, Koike I. 1998. Direct Determination of Carbon and Nitrogen Contents of Natural Bacterial Assemblages in Marine Environments. Applied and Environmental Microbiology 64:3352-3358.

13. Klindworth A, Pruesse E, Schweer T, Peplies J, Quast C, Horn M, Glöckner FO. 2013. Evaluation of general 16S ribosomal RNA gene PCR primers for classical and next-generation sequencing-based diversity studies. Nucleic Acids Research 41:e1.

14. Martin M. 2011. Cutadapt removes adapter sequences from high-throughput sequencing reads. EMBnetjournal 17:10-12.

15. Callahan BJ, McMurdie PJ, Rosen MJ, Han AW, Johnson AJA, Holmes SP. 2016. DADA2: High-resolution sample inference from Illumina amplicon data. Nat Meth 13:581-583.

16. Yutin N, Suzuki MT, Béjà O. 2005. Novel Primers Reveal Wider Diversity among Marine Aerobic Anoxygenic Phototrophs. Applied and Environmental Microbiology 71:8958-8962.

17. Dinno A. 2017. dunn.test: Dunn's Test of Multiple Comparisons Using Rank Sums. R package version 1.3.5. Accessed

18. Love MI, Huber W, Anders S. 2014. Moderated estimation of fold change and dispersion for RNA-seq data with DESeq2. Genome Biology 15:550.

19. McMurdie PJ, Holmes S. 2013. phyloseq: An R Package for Reproducible Interactive Analysis and Graphics of Microbiome Census Data. PLOS ONE 8:e61217.

20. Wickham H. 2009. ggplot2: Elegant graphics for data analysis. Springer-Verlag New York.

21. Auguie B. 2017. gridExtra: Miscellaneous Functions for "Grid" Graphics. Accessed
